# Mapping the signaling network of BIN2 kinase using TurboID-mediated biotin labeling and phosphoproteomics

**DOI:** 10.1093/plcell/koad013

**Published:** 2023-01-20

**Authors:** Tae-Wuk Kim, Chan Ho Park, Chuan-Chih Hsu, Yeong-Woo Kim, Yeong-Woo Ko, Zhenzhen Zhang, Jia-Ying Zhu, Yu-Chun Hsiao, Tess Branon, Krista Kaasik, Evan Saldivar, Kevin Li, Asher Pasha, Nicholas J Provart, Alma L Burlingame, Shou-Ling Xu, Alice Y Ting, Zhi-Yong Wang

**Affiliations:** Department of Plant Biology, Carnegie Institution for Science, Stanford, California 94305, USA; Department of Life Science, Hanyang University, Seoul 04763, South Korea; Research Institute for Convergence of Basic Science, Hanyang University, Seoul 04763, South Korea; Department of Plant Biology, Carnegie Institution for Science, Stanford, California 94305, USA; Department of Plant Biology, Carnegie Institution for Science, Stanford, California 94305, USA; Institute of Plant and Microbial Biology, Academia Sinica, Taipei 11529, Taiwan; Department of Life Science, Hanyang University, Seoul 04763, South Korea; Department of Life Science, Hanyang University, Seoul 04763, South Korea; Department of Plant Biology, Carnegie Institution for Science, Stanford, California 94305, USA; Department of Plant Biology, Carnegie Institution for Science, Stanford, California 94305, USA; Department of Plant Biology, Carnegie Institution for Science, Stanford, California 94305, USA; Departments of Genetics, Biology, and Chemistry, Stanford University, Stanford, California 94305, USA; Department of Biology, Stanford University, Stanford, California 94305, USA; Massachusetts Institute of Technology, Cambridge, Massachusetts 02139, USA; Department of Pharmaceutical Chemistry, University of California, San Francisco, California 94158, USA; Department of Plant Biology, Carnegie Institution for Science, Stanford, California 94305, USA; Department of Biology, Stanford University, Stanford, California 94305, USA; Department of Plant Biology, Carnegie Institution for Science, Stanford, California 94305, USA; Department of Cell & Systems Biology/Centre for the Analysis of Genome Evolution and Function, University of Toronto, Toronto, Ontario M5S 3B2, Canada; Department of Cell & Systems Biology/Centre for the Analysis of Genome Evolution and Function, University of Toronto, Toronto, Ontario M5S 3B2, Canada; Department of Pharmaceutical Chemistry, University of California, San Francisco, California 94158, USA; Department of Plant Biology, Carnegie Institution for Science, Stanford, California 94305, USA; Departments of Genetics, Biology, and Chemistry, Stanford University, Stanford, California 94305, USA; Department of Biology, Stanford University, Stanford, California 94305, USA; Chan Zuckerberg Biohub, San Francisco, California, USA; Department of Plant Biology, Carnegie Institution for Science, Stanford, California 94305, USA

## Abstract

Elucidating enzyme–substrate relationships in posttranslational modification (PTM) networks is crucial for understanding signal transduction pathways but is technically difficult because enzyme–substrate interactions tend to be transient. Here, we demonstrate that TurboID-based proximity labeling (TbPL) effectively and specifically captures the substrates of kinases and phosphatases. TbPL-mass spectrometry (TbPL-MS) identified over 400 proximal proteins of *Arabidopsis thaliana* BRASSINOSTEROID-INSENSITIVE2 (BIN2), a member of the GLYCOGEN SYNTHASE KINASE 3 (GSK3) family that integrates signaling pathways controlling diverse developmental and acclimation processes. A large portion of the BIN2-proximal proteins showed BIN2-dependent phosphorylation in vivo or in vitro, suggesting that these are BIN2 substrates. Protein–protein interaction network analysis showed that the BIN2-proximal proteins include interactors of BIN2 substrates, revealing a high level of interactions among the BIN2-proximal proteins. Our proteomic analysis establishes the BIN2 signaling network and uncovers BIN2 functions in regulating key cellular processes such as transcription, RNA processing, translation initiation, vesicle trafficking, and cytoskeleton organization. We further discovered significant overlap between the GSK3 phosphorylome and the *O*-GlcNAcylome, suggesting an evolutionarily ancient relationship between GSK3 and the nutrient-sensing *O*-glycosylation pathway. Our work presents a powerful method for mapping PTM networks, a large dataset of GSK3 kinase substrates, and important insights into the signaling network that controls key cellular functions underlying plant growth and acclimation.

## Teaser

Combining TurboID-mediated proximity labeling with quantitative phosphoproteomics identifies BIN2 signaling components including kinase substrates in vivo, revealing cellular functions of BIN2.

**IN A NUTSHELL**

**Background:** Intracellular signal transduction relies on specific and dynamic interactions between kinases and their substrates. Identifying substrate proteins of each kinase is crucial for understanding cellular signaling transduction pathways but is technically challenging because of the transient nature of the enzyme–substrate interactions and the large number of kinases acting in a cell. *Arabidopsis thaliana* BRASSINOSTEROID-INSENSITIVE2 (BIN2) is one of the best-studied plant kinases, with key roles in multiple signaling pathways including the brassinosteroid and auxin pathways. However, BIN2's in vivo interactors and substrate proteins have not been fully characterized. Recent studies have developed the TurboID biotin ligase as a highly efficient proximity labeling tool; its efficiency in mapping transient protein–protein interactions has not been fully explored.

**Question:** Can a fusion protein containing a kinase and the TurboID biotin ligase biotinylate the substrate proteins phosphorylated by the BIN2 kinase? Is this approach effective, when combined with phosphoproteomics, in identifying kinase substrates that interact transiently? What are the substrates and cellular targets of BIN2? How does the BIN2 signaling network overlap with other signaling pathways?

**Findings:** We show that TurboID is an effective and specific tool for mapping kinase signaling networks. We identified 482 BIN2-proximal proteins, including about two-thirds that showed BIN2-dependent phosphorylation and many known BIN2 interactors and substrates. The dataset of in vivo BIN2 interactors and substrates uncovers an expansive signaling network and reveals a convergence between the BIN2/GSK3 and *O*-GlcNAc modification pathways in both plants and animals.

**Next steps:** How BIN2 acts specifically in various signaling pathways and how it regulates various substrate proteins and cellular functions are key questions to be answered in future studies. How BIN2-mediated phosphorylation crosstalks with *O*-GlcNAcylation is another important question with broad implications. Our dataset of candidate proteins with modification sites will enable future investigations that advance our understanding of these important questions.

## Introduction

GLYCOGEN SYNTHASE KINASE 3 (GSK3) is a major signaling hub in animals and plants (Youn and Kim, 2015; Patel and Woodgett, 2017; Li et al., 2021). In animals, GSK3 was initially identified as a regulator of sugar metabolism but has since been found to act in numerous signaling pathways and phosphorylate over a hundred cellular proteins. As a hub of cellular signal transduction, GSK3 has been implicated in major human diseases such as diabetes, cancer, and neurological disorders (Beurel et al., 2015; Patel and Woodgett, 2017). In plants, the best-characterized GSK3-like kinase, BRASSINOSTEROID-INSENSITIVE2 (BIN2), was first identified as a key component of the brassinosteroid (BR) signaling pathway in *Arabidopsis thaliana*, where BIN2 phosphorylates and inhibits the BRASSINAZOLE-RESISTANT1 (BZR1) family of BR-responsive transcription factors (Li and Nam, 2002). BR signaling through the receptor kinase BRASSINOSTEROID INSENSITIVE1 (BRI1) leads to BIN2 dephosphorylation, ubiquitination, and degradation, resulting in protein phosphatase 2A (PP2A)-mediated dephosphorylation and nuclear accumulation of BZR1, which mediate BR-responsive gene expression and plant growth (He et al., 2002; Kim and Wang, 2010; Tang et al., 2011; Zhu et al., 2017).

Recent molecular genetic studies have elucidated BIN2's broad functions in a wide range of developmental and physiological processes (Youn and Kim, 2015; Li et al., 2021). For example, BIN2 acts in additional receptor kinase pathways that regulate asymmetric cell division, differentiation of stomata and xylem cells, and development of lateral roots (Kim et al., 2012; Cho et al., 2014; Kondo et al., 2014; Youn and Kim, 2015; Houbaert et al., 2018; Li et al., 2021). BIN2 also interacts with components of other hormonal and light signaling pathways and plays a role in salt stress responses (Li et al., 2020, 2021). Identification of all BIN2 kinase substrates is crucial for understanding the cellular functions regulated by this signaling hub that integrates diverse regulatory pathways in plants.

Identification of in vivo kinase substrates is technically challenging because the interactions between a kinase, or any posttranslational modifying enzyme, and its substrate proteins need to be transient and dynamic in order to rapidly modify many substrate molecules. Traditional methods for identifying protein–protein interactions (PPI), such as co-immunoprecipitation (co-IP), tend to capture only stable interactors that remain associated throughout the incubation and washing procedures. The recent development of a highly active biotin ligase named TurboID has made it possible to effectively biotinylate and thus identify in vivo transient interactors (Branon et al., 2018; Samavarchi-Tehrani et al., 2020). TurboID has been used for proximity labeling (PL) of subcellular proteomes and interactomes of transcription factors and immune receptors in plants (Mair et al., 2019; Zhang et al., 2019). TurboID has also been shown to detect various PPI in several plant model systems (Arora et al., 2020). Here, we test the idea that TurboID fused to a kinase or phosphatase can biotinylate their substrate proteins while they are phosphorylated or dephosphorylated. We demonstrate that TurboID fused with BIN2 and PP2A specifically and effectively biotinylates their substrates. Mass spectrometry identified 482 proteins that are biotinylated by a BIN2-TurboID fusion protein; over one-third of these BIN2-proximal proteins showed dephosphorylation upon inhibition of BIN2 and thus are considered BIN2 substrates. Protein–protein interaction and protein function analyses assembled the BIN2-proximal proteins into signaling networks and illustrated BIN2 regulation of key cellular functions. Our study provides a powerful method for dissecting PTM networks and a large dataset of the BIN2 signaling network that reveals broad functions of this highly conserved signaling hub.

## Results

### TurboID fused to a kinase and phosphatase specifically biotinylates their substrates in vivo

**Figure 1:**
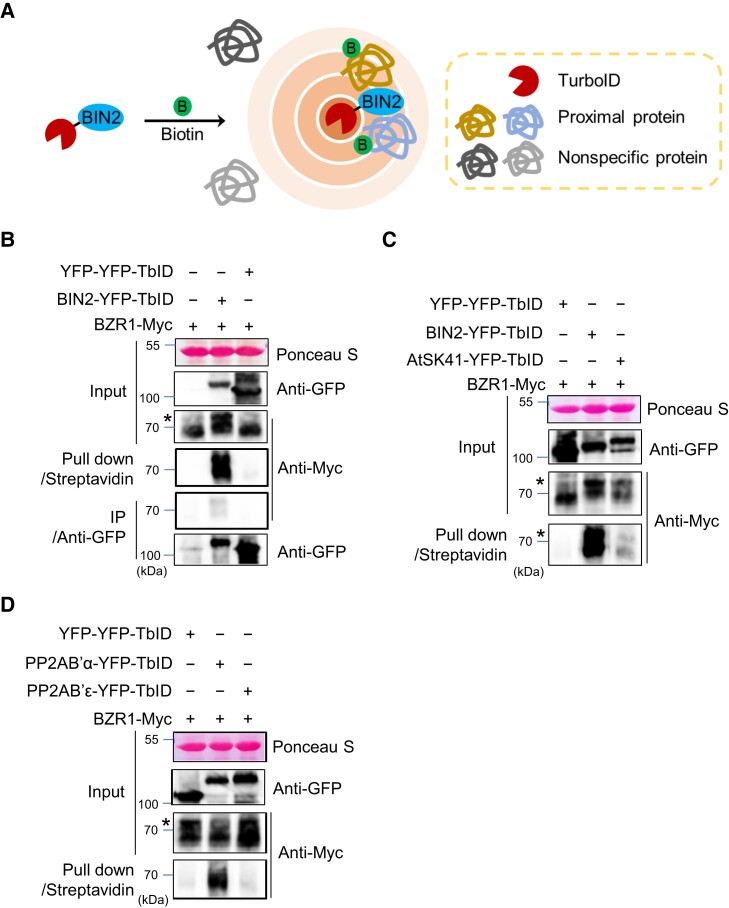
Biotinylation of BZR1 by BIN2-TurboID and PP2AB′α-TurboID. A, Schematic diagram of proximity-dependent biotin labeling. B, Biotinylation of BZR1 by proximity labeling. The indicated TurboID (TbID) fusion proteins were co-expressed with BZR1-Myc in *N. benthamiana* leaves. Streptavidin pulldown and anti-GFP IP were performed using aliquots of the same protein extracts, and proteins were immunoblotted using antibodies shown on the right side. Ponceau S staining shows the loading of the input. Streptavidin pulled down 10.4-fold more BZR1-Myc than anti-GFP IP. Asterisks indicate phosphorylated BZR1. C, Comparison of BZR1 biotinylation by BIN2-TbID and AtSK41-TbID. D, Comparison of BZR1 biotinylation by PP2AB′α-TbID and PP2AB′ɛ-TbID.

We created a DNA construct to express full-length BIN2 fused with YFP (yellow fluorescent protein) and TurboID (BIN2-YFP-TbID) from the constitutive 35S promoter (Supplemental Figure 1; Figure 1A). To test whether BIN2-YFP-TbID can biotinylate BZR1, the canonical BIN2 substrate in the BR signaling pathway, we co-expressed BIN2-YFP-TbID with 35S:BZR1-MH (BZR1 fused with 4×Myc-6×His tag) in *Nicotiana benthamiana* leaves. As a control, we also co-expressed BZR1-MH with a YFP-YFP-TbID fusion protein. After pulldown by streptavidin-agarose from the protein extracts, immunoblotting with anti-Myc antibody detected BZR1-MH in the sample co-expressing BIN2-YFP-TbID but not in the control expressing BZR1-MH alone or co-expressing YFP-YFP-TbID (Figure 1B), indicating that BIN2-YFP-TbID specifically caused biotinylation of BZR1. From the same extract, the streptavidin beads pulled down about 10 times more BZR1-MH than did the anti-GFP antibody beads, indicating a higher sensitivity of TbPL than co-IP for detecting in vivo PPI (Figure 1B).

We further examined the specificity of TurboID for biotinylation of interacting proteins. Previous molecular and genetic studies demonstrated that BZR1 interacts with six GSK3 kinases including BIN2 but not with three other GSK3s including SHAGGY-LIKE PROTEIN KINASE 41 (AtSK41) (Kim et al., 2009). We found that BZR1-MH was pulled down much more effectively by streptavidin when it was co-expressed with BIN2-YFP-TbID than when it was co-expressed with AtSK41-YFP-TbID (Figure 1C). Previous studies also showed that BZR1 binds to specific PP2A regulatory subunits such as PP2AB′α but not to PP2AB′ε (Tang et al., 2011). Biotinylation of BZR1-MH was detected when co-expressed with PP2AB′α-YFP-TbID but not when co-expressed with PP2AB′ε-YFP-TbID (Figure 1D). These results indicate that TurboID is a sensitive and specific method for identifying substrates of kinases and phosphatases.

### BIN2-TurboID biotinylates BR signaling components and hundreds of cellular proteins

**Figure 2:**
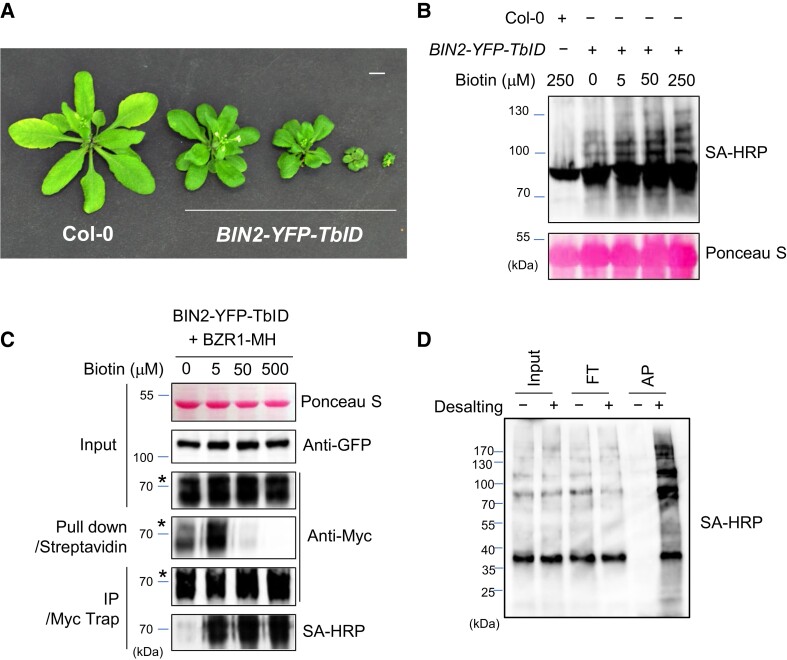
The effect of biotin concentration on the efficiency of biotin labeling and affinity purification in Arabidopsis. A, Phenotypes of the wild-type (Col-0) and transgenic Arabidopsis overexpressing *BIN2-YFP-TbID*. Plants were grown for 4 weeks in soil. The scale bar is 1 cm. B, The effect of biotin concentration on TbID-mediated biotinylation. 14-d-old BIN2-YFP-TbID seedlings were treated with the indicated concentrations of biotin for 1 h. Total proteins were immunoblotted using streptavidin-HRP (SA-HRP). C, The effect of biotin concentration on biotinylation and streptavidin pulldown of BZR1. D, The effect of desalting on affinity purification of biotinylated proteins. *BIN2-YFP-TbID* seedlings were treated with 50 mM biotin for 3 h. Equal aliquots of protein extracts were desalted (+) or not desalted (−) before streptavidin pulldown. Input (1:300), flow through (FT, 1:300) and eluate after affinity pulldown (AP, 1:10) were separated by SDS-PAGE. Biotinylated proteins were probed with SA-HRP.

To identify BIN2-proximal proteins, we generated transgenic Arabidopsis (*Arabidopsis thaliana*) plants expressing BIN2-YFP-TbID, or YFP-YFP-TbID as a control, from the 35S promoter. Some of the plants expressing *BIN2-YFP-TbID* displayed similar dwarf phenotypes to those observed in the BIN2-overexpressing or *bin2-1* mutant plants (Li and Nam, 2002), whereas plants expressing YFP-YFP-TbID were indistinguishable from the wild-type (Figure 2A; Supplemental Figure 2A), suggesting that BIN2-YFP-TbID is functional in Arabidopsis and that TurboID has no obvious effect on BIN2 or other plant functions. BIN2-YFP-TbID and YFP-YFP-TbID showed similar subcellular localization (Supplemental Figure 2B; Kim et al., 2009). We chose *BIN2-YFP-TbID* transgenic lines that showed visible but subtle phenotypes, which indicate moderate expression levels, for PL experiments (Supplemental Figure 2, A and C). Reverse transcription quantitative PCR (RT-qPCR) showed that the *BIN2-YFP-TbID* plants expressed about 4-fold more *BIN2* RNA than wild-type plants (Supplemental Figure 2D). However, a smaller increase is expected for BIN2 protein, which is posttranslationally regulated by ubiquitination and degradation (Zhu et al., 2017). Treating *BIN2-YFP-TbID* tissues with biotin caused protein biotinylation in a dose-dependent manner (Figure 2B). However, a high concentration of biotin decreased the efficiency of streptavidin pulldown of biotinylated proteins, potentially due to competition by free biotin (Figure 2C). Indeed, removing free biotin using a desalting column recovered streptavidin pulldown efficiency (Figure 2D).

**Figure 3:**
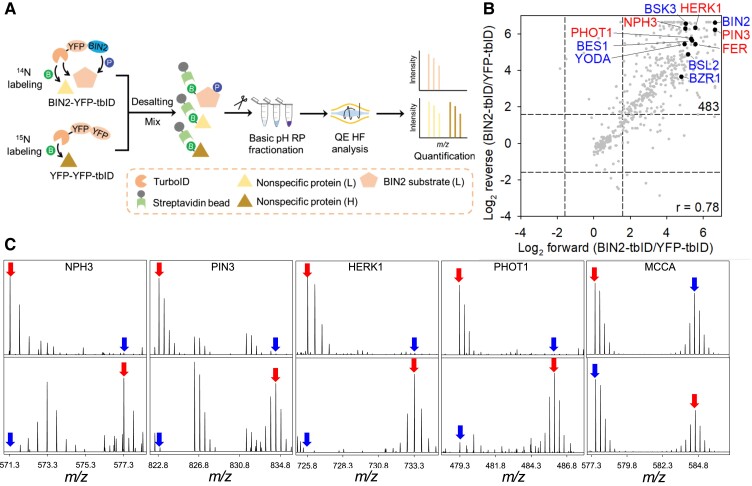
TurboID-based identification of BIN2-proximal proteins in Arabidopsis. A, Schematic diagram of the workflow of isotope labeling, proximity biotinylation (B) while BIN2 phosphorylates (P) its substrates, purification, fractionation by reverse phase chromatography (RP), and analysis on mass spectrometer (QE-HF) of BIN2-proximal proteins. B, Signal ratios between BIN2-YFP-TbID and YFP-YFP-TbID for proteins detected in two replicate experiments where isotopes were switched. Blue colored letter indicates previously reported BIN2 interactors. The dashed lines show a 3-fold cutoff ratio. C, Representative MS1 spectra show the enrichment of NPH3, PIN3, HERK1, PHOT1, and no enrichment of MCCA, by BIN2-YFP-TbID relative to the YFP-YFP-TbID control. Top panel: ^14^N: BIN2-YFP-TbID, ^15^N: YFP-YFP-TbID; Bottom panel: ^14^N: YFP-YFP-TbID, ^15^N: BIN2-YFP-TbID. Red and blue arrows point to the monoisotopic peaks of BIN2-YFP-TbID samples and YFP-YFP-TbID control, respectively.

To quantitatively distinguish the BIN2-proximal proteins from those tagged non-specifically by the YFP-YFP-TbID control, we used stable isotope labeling in Arabidopsis-mass spectrometry (SILIA-MS) (Figure 3A). We grew *BIN2-YFP-TbID* and *YFP-YFP-TbID* seedlings on medium containing either a ^14^N or ^15^N nitrogen source for 16 days, mixed equal amounts of the two tissues together and then treated them with 50 µM biotin for 3 h. The protein extract was processed through a desalting column to remove free biotin, and the biotinylated proteins were affinity-purified using streptavidin beads and then digested with trypsin on the beads. The digested peptides were fractionated and analyzed by liquid chromatography-tandem mass spectrometry (LC-MS/MS) (Figure 3A). The experiment was repeated four times with the isotopes switched (the *BIN2-YFP-TbID* sample was labeled with ^15^N in two biological replicates and the *YFP-YFP-TbID* control was ^15^N-labeled in the other two replicates). Quantitation based on isotope ratios showed that BIN2 and 482 proteins were enriched over 3-fold in BIN2-YFP-TbID compared with in the YFP-YFP-TbID control in at least three of the four replicates, and these were considered BIN2-proximal proteins (Figure 3, B–C; Supplemental Dataset 1).

The BIN2-proximal proteins include many components of the BR signaling pathway including BRI1 SUPPRESSOR 1 (BSU1)-LIKE 1 (BSL1), BSL2, BSL3, BRASSINOSTEROID-SIGNALING KINASE3 (BSK3), PP2A, TOPLESS (TPL), and its homologs (TPL-related: TPR1, TPR2, TPR4) (Oh et al., 2014), as well as known BIN2 substrates including BZR1, BZR2/BRI1-EMS-SUPPRESSOR 1 (BES1), cellulose synthase (Sanchez-Rodriguez et al., 2017), and the MAPK kinase kinase YODA (YDA) (Kim et al., 2012). Additional BIN2-proximal proteins that are involved in known BR functions include the auxin transporters PIN-FORMED 3, 4, 7 (PIN3), PIN4, PIN7), the HERCULES (HERK1), and FERONIA (FER) receptor kinases involved in cell wall integrity pathways, the blue-light photoreceptor PHOTOTROPIN1 (PHOT1), and PHOT1 interacting protein NONPHOTOTROPIC HYPOCOTYL3 (NPH3) involved in phototropism (Figure 3, B–C).

### Quantitative phosphoproteomics identifies BIN2-dependent phosphoproteins among BIN2-proximal proteins

**Figure 4:**
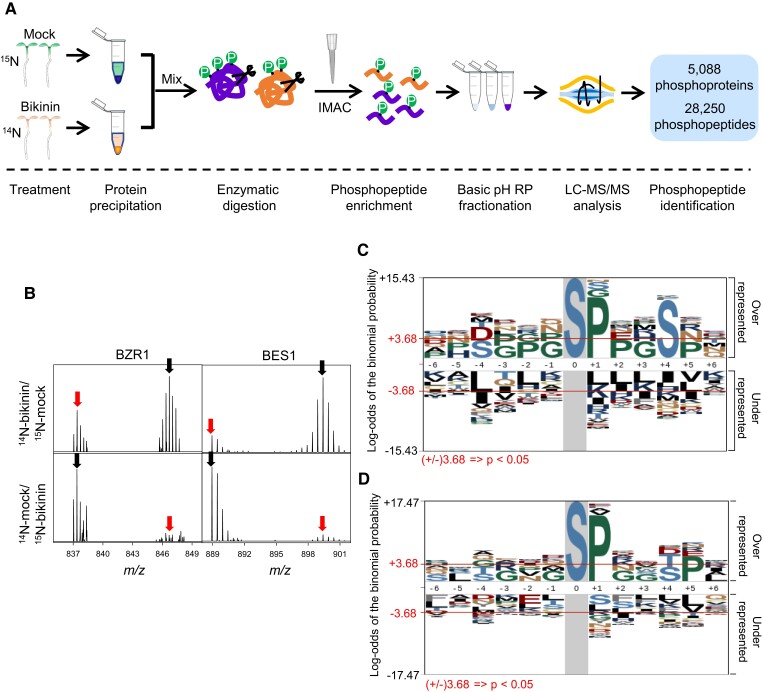
Identification of bikinin-responsive phosphoproteins. A, Schematic diagram of phosphoproteomic analysis of bikinin responses using stable isotope labeling mass spectrometry. The seedlings were treated with mock or bikinin (30 µM) for 1 h. P and IMAC indicate phosphate and immobilized metal affinity chromatography, respectively. B, Representative MS1 spectra show the signals of phosphopeptides of BZR1 and BES1 in bikinin- (red arrow) and mock-treated (black arrow) samples. Top and bottom panels are repeats experiments with isotope reversed. C and D, Phosphorylation motifs enriched in the phosphopeptides decreased (C) or increased (D) by bikinin treatment.

To identify the substrates of BIN2 kinase among the BIN2-proximal proteins, we performed quantitative phosphoproteomic analysis after treatment of Arabidopsis with bikinin, a specific inhibitor of BIN2 and its homologous GSK3 kinases (De Rybel et al., 2009; Figure 4A). After growth on ^14^N or ^15^N media (isotopes reversed in a repeat experiment) for 14 days, the tissues were treated with 30 µM bikinin or mock solution for 1 h. The effect of bikinin was confirmed by immunoblotting showing BZR1 dephosphorylation and RT-qPCR showing the expected changes of expression of the BR-repressed BZR1-target gene *DWARF4* (*DWF4*) and BR-induced *SMALL AUXIN UP RNA 19 (SAUR19)* (Supplemental Figure 3, A–C). The pair of bikinin- and mock-treated isotope-labeled samples were mixed and the phosphopeptides were enriched by immobilized metal affinity chromatography (IMAC). LC-MS/MS analysis identified a total of 28,250 phosphopeptides from 5,088 phosphoproteins in two repeat experiments in which the isotopes were reversed between the bikinin and mock treatments (Figure 4A). Among these, 741 phosphoproteins (14.6%) showed a decrease of at least one phosphopeptide upon bikinin treatment in the two reverse-labeling replicates (Supplemental Dataset 2). These included the known BIN2 substrates BZR1 and BZR2/BES1 (Figure 4B). Of the 482 BIN2-proximal proteins, 410 were detected as phosphoproteins, and 169 (41.2%) of these showed dephosphorylation after bikinin treatment (Supplemental Datasets 1 and 2).

GSK3 kinases are known to phosphorylate substrates at consensus recognition motifs of S/TxxxS/T, where S and T are serine and threonine, and x is any amino acid (Beurel et al., 2015). Motif analysis of the identified phosphorylation sites showed that the consensus GSK3 phosphorylation motif is significantly over-represented among the bikinin-decreased phosphopeptides of the 169 BIN2 substrates, but not in the phosphopeptides increased by bikinin treatment (Figure 4, C and D), consistent with evolutionary conservation of GSK3 phosphorylation sites.

**Figure 5:**
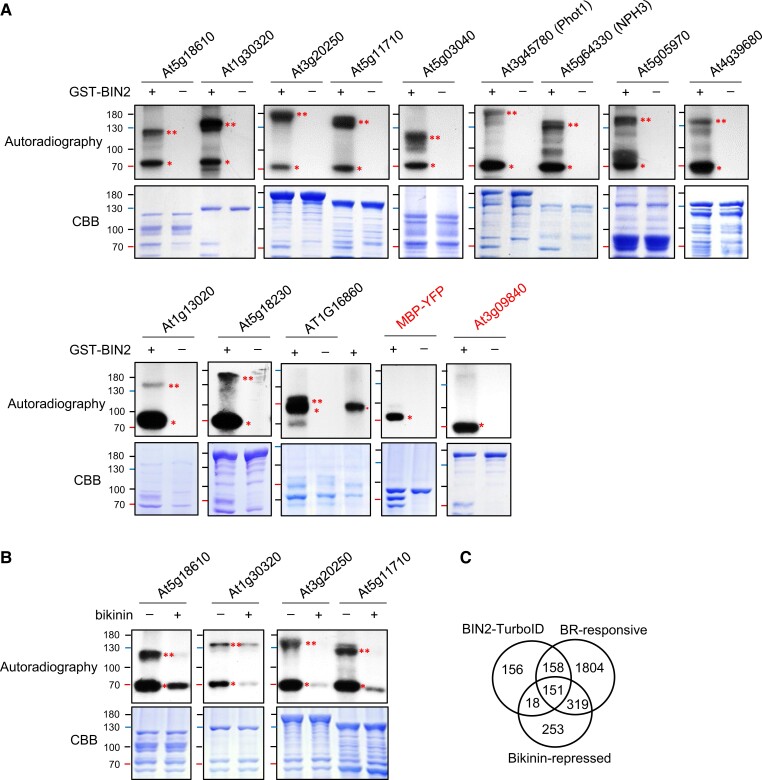
Validation of BIN2 phosphorylation of putative substrate proteins. A, In vitro kinase assays of GST-BIN2 and MBP-fused substrates to test BIN2 phosphorylation of the BIN2 substrates. MBP-fused protein of At5g18610 (kinase-inactive form; K112R), At1g30320, At3g20250, At5g11710, At5g03040, At3g45780 (kinase-inactive form; D806N), At5g64330, At1g13020, At5g18230, At5g05970, At4g39680, or At1g16860 (N-terminal partial protein; 1-209 aa) was incubated with GST-BIN2 in a kinase buffer containing ^32^P-γ-ATP. MBP-YFP and MBP-fused At3g09840 were used as negative controls. CBB, Coomassie Brilliant Blue. B, Inhibition of BIN2 phosphorylation by bikinin. Four MBP-fused proteins were incubated with GST-BIN2 in a kinase assay buffer without or with 15 μM bikinin. Single and double asterisks indicate the auto-phosphorylated GST-BIN2 and the substrate proteins phosphorylated by GST-BIN2, respectively. C, Venn diagram shows overlaps among BIN2-proximal proteins (BIN2-TurboID), bikinin-repressed, and BR-responsive phosphoproteins (Clark et al., 2021).

To test how likely these bikinin-sensitive BIN2-proximal phosphoproteins are substrates of BIN2 kinase, we expressed 12 of these proteins as fusions to the maltose-binding protein (MBP) in *E. coli*, affinity-purified the proteins, and performed in vitro kinase assays. The results show that all 12 proteins can be phosphorylated by BIN2 in vitro, whereas MBP fused to YFP or a non-BIN2-proximal protein (At3g09840) was not phosphorylated by BIN2 (Figure 5A; Supplemental Figure 4). As expected, bikinin inhibited the phosphorylation in vitro (Figure 5B), as it did in vivo. These results indicate that the BIN2-proximal proteins that showed dephosphorylation upon bikinin treatment are mostly substrates of BIN2 kinase.

BIN2 is inactivated and degraded upon BR signaling (Zhu et al., 2017) and thus its substrates and interactors in the BR pathway are expected to respond to BR. A recent proteomic study identified 2432 BR-responsive phosphoproteins (Clark et al., 2021). These included 309 (64%) of the 482 BIN2-proximal proteins and 151 (89%) of the 169 bikinin-sensitive BIN2-proximal proteins. Together, 327 (68%) of the 482 BIN2-proximal phosphoproteins changed phosphorylation level in response to BR and/or bikinin treatments (Figure 5C).

### The BIN2 signaling network

**Figure 6:**
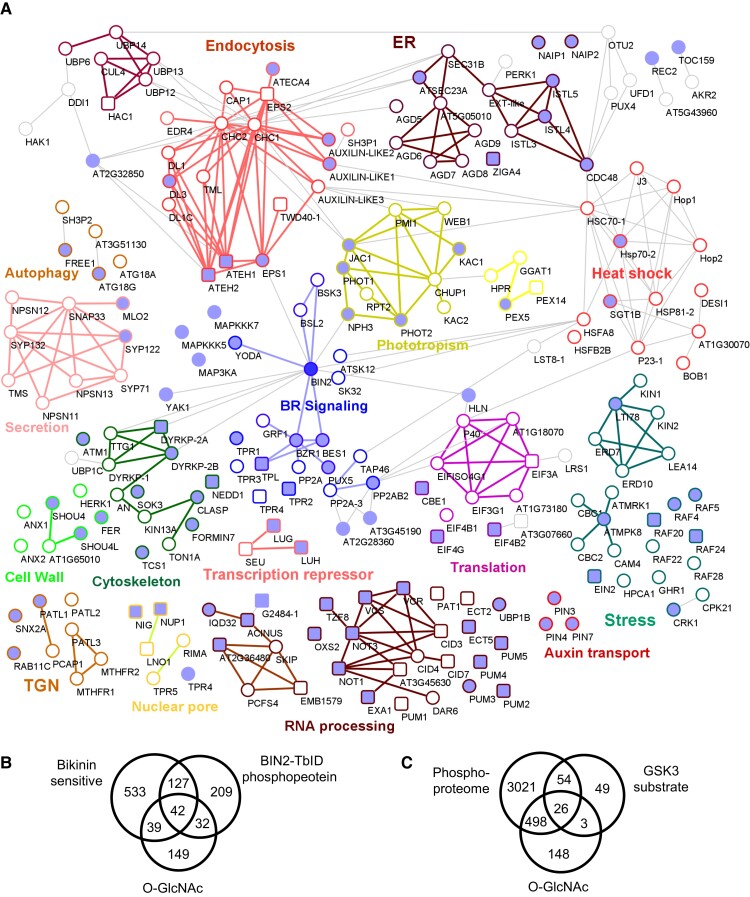
The BIN2 signaling network. A, The diagram of the BIN2 signaling network shows BIN2 substrates and their interactors. The filled nodes (circles and squares) represent BIN2 substrates identified as BIN2-proximal proteins that showed dephosphorylation upon bikinin treatment. The rectangle nodes indicate *O*-GlcNAcylated proteins. Clusters with specific biological functions are highlighted by different colors of edges. ER, Endoplasmic Reticulum, BR, Brassinosteroid, TGN, Trans-Golgi Network. B, Venn diagram shows the overlaps among the bikinin-sensitive phosphoproteins, BIN2-proximal phosphoproteins (BIN2-TbID), and *O*-GlcNAcylated proteins (Xu et al., 2017) in Arabidopsis. C, Venn diagram shows the overlaps among the phosphoproteins and *O*-GlcNAcylated proteins in synapse and GSK3 substrates in the hippocampus of mouse (Trinidad et al., 2012; Kaasik et al., 2013).

We constructed a BIN2 signaling network by combining our proximity-tagging and phosphoproteomic datasets with protein interaction databases (Figure 6A). Based on the PPI established by experimental evidence (Dong et al., 2019; Szklarczyk et al., 2019), 98 BIN2-proximal proteins that showed no phosphorylation change upon bikinin treatment are interactors of some of the bikinin-sensitive BIN2-poximal proteins, which we consider BIN2 substrates. Together, the BIN2 substrates and their interactors account for 267 (55%) of the 482 BIN2-proximal proteins identified by BIN2-TurboID (Figure 6A).

The proximity-based BIN2 signaling network includes a large number of known upstream regulators (BSK3, BSL1, BSL2, BSL3, OCTOPUS LIKE 2), BIN2 homologs (AtSK12 and AtSK31), BIN2 substrates (BZR1, BES1, and YDA), and interactors of BZR1 (five PP2A components, all five members of the TPL/Groucho/TLE co-repressor family, and a 14-3-3 protein) (Kim and Wang, 2010; Kim et al., 2012; Oh et al., 2014; Anne et al., 2015; Figure 6A). The BIN2 substrates overrepresent several key cellular functions, including transcription repression, RNA processing, translation initiation, vesicle trafficking, cytoskeleton organization, cell wall integrity, and phototropic responses. The large number of BIN2 substrates indicates that GSK3 orchestrates broad cellular and developmental processes in plants.

### GSK3 and *O*-GlcNAc target overlapping proteomes in both Arabidopsis and animals

Our identification of the GSK3 phosphorylome in Arabidopsis provides an opportunity to understand the relationship of GSK3 with other signaling pathways in the cellular regulatory system. We found a striking level of overlap between the BIN2 phosphorylome and the *O*-GlcNAcylome, proteins modified by *O*-linked β-*N*-acetylglucosamine (*O*-GlcNAc). *O*-GlcNAc modification of nucleocytoplasmic proteins is an essential nutrient-sensing mechanism known to regulate cellular homeostasis in animals as well as growth and development in plants (Hart, 2019; Sun, 2021). The 262 *O*-GlcNAc modified proteins identified in Arabidopsis (Xu et al., 2017) include 42 (25%) of the 169 BIN2 substrates, 22 (14%) of the 158 BR-responsive BIN2-proximal proteins, and only 11 (7%) of the remaining 156 BIN2-proximal proteins that did not show responses to bikinin or BR (Figure 6B; Supplemental Dataset 1). Additionally, 46 *O*-GlcNAcylated proteins showed a bikinin-induced decrease in phosphorylation but were not detected as BIN2-proximal proteins. As such, about 46% of the *O*-GlcNAcylated proteins showed either GSK3-dependent phosphorylation or were in proximity to BIN2, or both. These over represent a small number of pathways including RNA processing, translation, and transcription. For example, *O*-GlcNAc modifies 13 of the 15 BIN2 substrates involved in RNA processing. Four of these 13 proteins contain peptides that were detected as modified by *O*-GlcNAc and BIN2 phosphorylation (Supplemental Figure 6), suggesting that the two types of PTMs on the same or nearby sites may influence each other. These observations suggest a functional crosstalk between the BIN2-catalyzed phosphorylation and *O*-GlcNAcylation of common target proteins.

Crosstalk between GSK3 and *O*-GlcNAc has been observed on several shared substrates in metazoans (Hart et al., 2011). These include c-Myc, c-Jun, β-catenin, tau, and α-synuclein, which play important roles in prevalent human diseases such as cancer and neuronal disorders (Yu et al., 2012; Ha et al., 2014). Phosphorylation and *O*-GlcNAcylation are known to crosstalk antagonistically on these proteins (Hart et al., 2011; Leney et al., 2017). To determine whether in animals, like in Arabidopsis, there is a significant overlap between substrates of GSK3 and *O*-GlcNAcylation at the proteomic level, we compared the 675 *O*-GlcNAcylated proteins identified in synapses with the 132 putative GSK3 substrates identified in the hippocampus in mouse (Trinidad et al., 2012; Kaasik et al., 2013). We found that 29 (22%) of the putative GSK3 substrates were identified also as *O*-GlcNAcylated proteins. Furthermore, among the 132 GSK3 substrates identified in the hippocampus, 80 were detected as phosphoproteins in the synapse, and 26 (33%) of these are *O*-GlcNAc modified proteins (Figure 6C; Supplemental Dataset 3). Such a significant overlap between substrates of *O*-GlcNAcylation and GSK3-mediated phosphorylation in the synapse suggests a broad role for the crosstalk between these two pathways in neuronal functions.

## Discussion

GSK3 is a conserved signaling hub that regulates diverse cellular, developmental, and adaptive processes in all eukaryotes. GSK3s have been studied extensively for their important roles in human health and plant growth. However, the complete GSK3 signaling network has not been illustrated in any organism. Taking advantage of the highly sensitive TbPL-MS approach, we here provide the largest GSK3 signaling network based on in vivo data and demonstrate that combining TbPL-MS with phosphoproteomics is a powerful approach to mapping PTM signaling networks. Our study uncovers broad GSK3 functions in cellular regulation and reveals the convergence of the GSK3 and *O*-GlcNAc pathways as a conserved feature of eukaryotic cellular regulation.

### PL-MS combined with quantitative PTM profiling is a powerful approach for mapping cellular regulatory pathways

PTM is the fundamental mechanism of cellular regulation. The relationships between the posttranslational modifying enzymes and their substrate proteins shape the cellular regulation network. Unlike many stable protein complexes, the interactions between modifying enzymes and their substrates in PTM networks tend to be dynamic and transient in order to allow rapid modification of multiple substrate molecules within a short time. The dynamic/transient nature of the interactions between a PTM enzyme and its substrates makes it difficult to identify these interactions using traditional methods such as affinity purification and co-IP, which capture only stable interactors that remain associated after the long procedure of extraction, incubation, and washing. In contrast, in PL-MS, the interacting proteins are tagged by biotin during the time of association in vivo, and then purified by streptavidin beads. In fact, the dynamic nature of the interactions that facilitate PTM would increase the efficiency of TurboID labeling compared with stable interactions, due to the amplification of signal from cycling interactions (Gingras et al., 2019). We believe that each kinase-TurboID fusion protein molecule can biotinylate, as well as phosphorylate, multiple copies of substrate proteins during the time of biotin treatment, allowing sensitive identification of the substrate proteins. Indeed, our direct comparison indicates that PL is about 10 times more sensitive than IP for detecting BZR1 as a BIN2 interactor, whereas similar IP-MS analyses identified only five putative BIN2-interactors (Supplemental Figure 5 and Supplemental Dataset 4), of which only one responded to bikinin or BR treatment.

The high sensitivity of detection raises the question of specificity. We provide several lines of evidence supporting the high specificity of the TbPL-MS approach for mapping kinase signaling pathways. First, we show that BZR1 is biotinylated much more strongly, if not exclusively, by the members of GSK3 and PP2A that are known to interact with BZR1 compared with their homologs that are known to not interact with BZR1. Second, TbPL-MS identified many known BIN2 substrates (e.g. BZR1, BES1, YDA, and CELLULOSE SYNTHASE 3 [CESA3]) and regulators (BSK3, BSL1, BSL2, BSL3, PP2A, and OCTOPUS LIKE 2 [OPL2]). Third, and most importantly, a large portion of the BIN2-proximal proteins identified by TbPL-MS showed dephosphorylation upon inhibition of BIN2. Of the 482 BIN2-proximal proteins, 169 (35%) showed bikinin-induced dephosphorylation in our phosphoproteomic analysis and 108 (43 additional) did so in a recent study (Lu et al., 2022), and 309 (64%) responded to BR treatment (Clark et al., 2021). Our in vitro kinase assays confirmed BIN2 phosphorylation of all twelve tested proteins. Montes et al. (2022) recently carried out in vitro BIN2 phosphorylation of total Arabidopsis protein extracts followed by phosphoproteomic analysis and showed BIN2 phosphorylation of 1,343 peptides from 767 proteins (Montes et al., 2022); these in vitro substrates include 104 BIN2-proximal proteins (Supplemental Dataset 1). Together, these studies provide evidence for BIN2-dependent phosphorylation of 344 (71%) of the BIN2-proximal proteins; 216 (45%) of these are supported by at least two of these studies and can be considered high-confidence BIN2 substrates (Supplemental Dataset 1).

The enrichment of the GSK3 consensus phosphorylation site (S/TxxxS/T) in these BIN2 substrates identified by PL indicates that the substrate specificity of plant GSK3s involves both physical proximity and the local sequence of the substrate proteins, raising questions about substrate identification based on in vitro phosphorylation conditions that disrupt the in vivo protein spatial organization. Among the 767 in vitro BIN2 substrates identified by Montes (Montes et al., 2022), 27.5% (211) showed dephosphorylation upon bikinin treatment (Supplemental Dataset 2; Lu et al., 2022), in contrast to 46% (221) of the 482 BIN2-proximal proteins (Supplemental Figure 7). The evidence overall indicates that TbPL-MS has superior sensitivity and specificity for mapping kinase signaling networks. The overlaps among these datasets identify subsets of putative BIN2 substrates with different confidence levels and suggest different efficiency and specificity of these complementary methods (Supplemental Figure 7).

### The GSK3 signaling network in Arabidopsis

Our PL-MS analysis establishes the largest experimental dataset of in vivo substrates of a GSK3 kinase in any organism and provides an expanded view of the GSK3 functions in plants. The BIN2 substrates reveal broad functions of GSK3 in regulating major cellular processes including transcription, RNA processing, translation, vesicle trafficking, cytoskeleton, cell wall synthesis/integrity, and auxin transport. Proximity to many signaling proteins such as receptors and kinases provides evidence for GSK3 functions in additional signaling pathways. Many proximal or substrate proteins are consistent with known functions of BR or GSK3. For example, 18 BIN2-proximal proteins, including seven BIN2 substrates, are components of clathrin-mediated endocytosis, and nine BIN2-proximal proteins, including two substrates (SYNTAXIN OF PLANTS 122 [SYP122] and MILDEW RESISTANCE LOCUS O 2 [MLO2]), are involved in secretion. In addition, several proteins involved in autophagy (e.g. AUTOPHAGY-RELATED PROTEIN 1B [ATG1B], AUTOPHAGY 18 [ATG18] G [ATG18G)], and FYVE DOMAIN PROTEIN REQUIRED FOR ENDOSOMAL SORTING 1 [FREE1]) are targets of BIN2 signaling, consistent with the recent reports of BR effects on autophagy (Chi et al., 2020). The microtubule-associated protein CLASP was recently reported to act in a BR signaling negative feedback loop (Ruan et al., 2018) and mediate microtubule reorientation (Lindeboom et al., 2019); our results suggest that BIN2 phosphorylation may mediate BR regulation of CLASP and microtubule orientation. BR and BIN2 have been shown to regulate auxin transport (Retzer et al., 2019); our results show that BIN2 directly phosphorylates the auxin transporters (PIN3, 4, 7). BR has also been reported to modulate phototropic responsiveness (Whippo and Hangarter, 2005); our data indicate that BIN2 phosphorylates and/or interacts with the photoreceptors phototropin 1 and 2 (PHOT1, PHOT2) and their downstream components that mediate phototropic growth (NPH3, PIN3, PIN4, and PIN7) and chloroplast movement (J-DOMAIN PROTEIN REQUIRED FOR CHLOROPLAST ACCUMULATION RESPONSE 1 [JAC1], KINESIN LIKE PROTEIN FOR ACTIN BASED CHLOROPLAST MOVEMENT 1 [KAC1], CHLOROPLAST UNUSUAL POSITIONING 1 [CHUP1]) (Fankhauser and Christie, 2015; Figure 6A). Genetic studies have placed the BR signaling module in a homeostatic feedback loop controlling cell wall extensibility and integrity that involve also the mechano- and integrity-sensing receptor kinases FER, HERK, ANXUR1 (ANX1), and ANX2 (Hofte, 2015); the identification of these receptor kinases as BIN2-proximal proteins suggests that BIN2 may play a direct role in the crosstalk between these wall- and BR-sensing RKs. A recent study shows that sugar increases BIN2 phosphorylation of BZR1 (Zhang et al., 2021), and this may involve BIN2 proximity with the mitogen-activated protein kinase kinase kinase named SUGAR INSENSITIVE 8 (SIS8) (Supplemental Dataset 1), which is required for sugar responses and interacts with a UDP-glucosyltransferase (Huang et al., 2014). Future studies of these BIN2-proximal and substrate proteins will advance our understanding of the functions of the GSK3 signaling network in cellular and developmental regulation in plants.

The ten members of the Arabidopsis GSK3 family appear to play overlapping and redundant roles. Considering that the BIN2-TurboID protein was expressed from the constitutive 35S promoter, some of the BIN2-proximal proteins identified in this study may associate with BIN2 homologs in wild-type plants. Our choice of BIN2-TurboID plants with a mild dwarf phenotype, compared with the dominant *bin2-1* mutant (Li and Nam, 2002), should have minimized artifacts. On the other hand, our BIN2 PL-MS experiments did not detect some of the previously reported BIN2 substrates (Li et al., 2021). Apparently, the full BIN2 signaling network is yet to be uncovered, likely by combining PL-MS and phosphoproteomics with a cell-type specific analysis under various physiological conditions. Furthermore, future PL-MS analysis of all GSK3 family members, using the native promoter of each gene for expression in its loss-of-function mutant background, will be required to provide a more accurate picture of the overlapping sub-networks of all ten GSK3 kinases in Arabidopsis.

### GSK3 and *O*-GlcNAc target overlapping proteomes in plants and animals

Our observations of overlaps between the targets of GSK3 and *O*-GlcNAc in both Arabidopsis and mouse synapses suggest that the convergence of these two PTM pathways is an ancient mechanism of cellular regulation. Modification at serine and threonine residues of nucleocytoplasmic proteins by *O*-GlcNAc, catalyzed by *O*-GlcNAc transferase (OGT) using UDP-GlcNAc as a donor substrate, is considered a nutrient-sensing mechanism that regulates cellular homeostasis by responding to the metabolic status of sugars, amino acids, lipids, and nucleotides. *O*-GlcNAc modification has been studied extensively in mammals for its important roles in numerous diseases including cancer, diabetes, neuron degeneration, and immune disorders (Banerjee et al., 2016). In addition to altering protein conformation and interactions, *O*-GlcNAc modification can crosstalk with phosphorylation on the common target proteins by steric competition for occupancy at the same or proximal sites (Hart et al., 2011; Leney et al., 2017). However, the relationships between *O*-GlcNAc and specific kinase pathways are not fully understood (Leney et al., 2017). Several studies suggest a close interaction between *O*-GlcNAcylation and GSK3. For example, a few proteins with important roles in human diseases such as beta-catenin (cancer), tau (Alzheimer's disease), and α-synuclein (Parkinson's) are targets of both GSK3 and *O*-GlcNAc (Yu et al., 2012; Ha et al., 2014). Inhibition of GSK3 increased and decreased *O*-GlcNAcylation of different proteins (Wang et al., 2007), whereas *O*-GlcNAc affects GSK3 phosphorylation of heat shock factor 1. These observations of functional crosstalk were explained by the mutual modifications between OGT and GSK3: OGT is a substrate of GSK3beta (Kaasik et al., 2013) and *O*-GlcNAcylation inhibits GSK3beta activity (Kazemi et al., 2010). The extent to which GSK3 and OGT target common substrates has remained unclear at the proteomic scale. Our findings of significant substrate overlap between GSK3 and OGT in both Arabidopsis and the mouse synapse suggest that co-regulation by GSK3-mediated phosphorylation and OGT-mediated *O*-GlcNAcylation might be an ancient and fundamental aspect of cellular homeostasis in eukaryotes. It's conceivable that GSK3 and *O*-GlcNAc respond to different upstream signals, e.g. growth factors and nutrients, to co-regulate cellular nutrient homeostasis and growth.

GSK3 and *O*-GlcNAc are known to impact neurological disorders such as Alzheimer's and Parkinson's diseases through co-regulation of β-catenin, tau, and α-synuclein (Hur and Zhou, 2010; Yuzwa and Vocadlo, 2014; Wheatley et al., 2019; Lauretti et al., 2020; Muha et al., 2020). The additional shared substrates identified in this study may also contribute to the crosstalk between GSK3 and *O*-GlcNAc in neuronal functions and thus should be of interest for future research. For example, delta-catenin is a GSK3 substrate associated with intellectual disabilities (Lu et al., 2016; Ryu et al., 2019), and the function of its *O*-GlcNAcylation remains to be studied.

Compared with the extensive investigation in mammals, research on *O*-GlcNAc in plants is at a stage of infancy. Recent studies clarified the molecular functions of the two OGT homologs SECRET AGENT (SEC) and SPINDLY (SPY) as *O*-GlcNAc transferase and *O*-fucose transferase, respectively (Zentella et al., 2017). Genetic evidence indicates that SEC/*O*-GlcNAcylation and SPY/*O*-fucosylation have overlapping functions that are essential for viability as well as unique or opposite functions in specific pathways (Sun, 2021). The *O*-GlcNAcylome data from Arabidopsis shows large numbers of key regulatory proteins as targets of *O*-GlcNAc modification (Xu et al., 2017); how *O*-GlcNAc regulates the functions of these proteins remains largely to be investigated. The proteins targeted by both *O*-GlcNAc and BIN2 are nodes of junctions between these pathways and are thus of particular interest. These over represent a small number of pathways including RNA processing, translation, and transcription. Among these proteins targeted by both BIN2/GSK3 and *O*-GlcNAc is ACINUS, which was shown recently to be also modified by *O*-fucose and to play major roles in developmental transition and stress responses by modulating transcription and RNA alternative splicing (Bi et al., 2021). Additional shared substrates include translation initiation factor eIF4B2 and eIF4E-binding protein (CBE1). Notably, *O*-GlcNAcylation of eIF4GI in mammalian cells modulates stress granule dynamics and the translational switch in stress responses (Zhang et al., 2018). In addition, BIN2 and *O*-GlcNAc target three of the five members of the TOPLESS (TPL) and all three components of the LEUNIG (LUG) family transcriptional repressors. TPL and LUG represent two subgroups of Gro/TLE-like co-repressors (Causier et al., 2012). The mammalian orthologs of TLEs are known to require *O*-GlcNAcylation for their transcriptional repression function (Yang et al., 2002; Wu et al., 2014). Thus, the function of *O*-GlcNAcylation in transcriptional repression seems to be conserved in plants and mammals. While not known to phosphorylate Gro/TLE co-repressors in animals, GSK3 has been shown to phosphorylate X-linked inhibitor of apoptosis protein (XIAP), which monoubiquitinates TLE in the Wnt pathway (Ng et al., 2018). As such, the similarity between the BR and Wnt pathways seems to extend from GSK3 phosphorylation of transcription factors (BZR1 and β-catenin) to *O*-GlcNAc modification of co-repressors (TPL and TLE). Future studies of the common targets of GSK3 and *O*-GlcNAc will shed light on the mechanism and logic of crosstalk between these two key regulatory systems, which are apparently important for both human health and agricultural productivity.

## Materials and methods

### Plant materials and growth condition

*Nicotiana benthamiana* seeds were planted in soil (Pro-Mix, Premier Tech, Quakertown, PA) and grown for 4–5 weeks in a greenhouse under natural sunlight at 25°C. YFP-YFP-TbID and BIN2-YFP-TbID were overexpressed in Arabidopsis (*Arabidopsis thaliana*) Col-0 ecotype. Arabidopsis seedlings were grown on ½-strength Murashige and Skoog (½ MS) medium (PhytoTechnology Laboratories, Shawnee Mission, KS) containing 1% (w/v) sucrose and 0.8% phytoagar (Caisson Laboratories, East Smithfield, UT).

### Plasmids

To generate a Gateway-compatible 35S-YFP-TbID vector, PCR fragments obtained from TurboID-containing plasmid (V5-TbID-NES_pCDNA3, Addgene) and pEarleyGate101 vector were assembled by overlapping ends using Gibson assembly master mix (NEB, Ipswich, MA). TurboID was amplified with primers FP1 (5′-ATCCACCGGATCTAGAGGCAAGCCCATCCCCAAC-3′) and RP1 (5′-AACATCGTATGGGTAAGGCAGCTGCAGCTTTTCGG-3′), while the pEarleyGate101 vector (1) was amplified with primers FP2 (5′-TACCCATACGATGTTCCAGATTACGCTTAATTAA-3′) and RP2 (5′-CTTGCCTCTAGATCCGGTGGATCCC-3′). The coding sequences of YFP and BIN2 cloned in pENTR/SD/D-TOPO were subcloned into a Gateway-compatible 35S-YFP-TurboID by an LR reaction (Invitrogen, Carlsbad, CA).

### *Nicotiana benthamiana* infiltration

Agrobacterium was inoculated into 5 ml of LB medium and grown for 16 h at 28°C. Cultured cells harvested from 1 ml aliquot were resuspended with 2 ml of the induction media (10 mM MES pH 5.6, 10 mM MgCl_2_, and 150 μM Acetosyringone), mixed according to the combination of plasmids, and incubated for 1 h at room temperature. Cells were infiltrated into abaxial leaves of *N. benthamiana* using a 1-ml syringe. After 36∼40 h, leaves were harvested and kept at −80°C until use.

### Confocal microscopy

BIN2-YFP-TbID and YFP-YFP-TbID seedlings were grown on MS agar medium for 8 days. YFP fluorescence of root segments was visualized with an SP8 confocal microscope (Leica Microsystems, Heerbrugg, Germany).

### Reverse transcription quantitative PCR

Total RNA was extracted using a Spectrum Plant Total RNA kit (Sigma) and complementary DNA (cDNA) was synthesized using M-MLV reverse transcriptase (Fermentas). Quantitative PCR (qPCR) was carried out using a LightCycler 480 (Roche) and SYBR Green Master Mix (Bioline). PP2A (For: 5′-TATCGGATGACGATTCTTCGTGCAG-3′, Rev: 5′-GCTTGGTCGACTATCGGAATGAGAG-3′) was used as an internal control. DWF4 (For: 5′-GGTGATCTCAGCCGTACATTTGGA-3′, Rev: 5′-CCCCACGTCGAAAAACTACCACTTC-3′) and SAUR19 (For: 5′-ACGTCGTCTCAAGCAGCATCTATCA-3′, Rev: 5′-CCCACGTAAACCGGAAAATGACCTT-3′) expression levels were normalized by PP2A.

### Protein extraction and immunoblot analysis

Plant tissues were ground with liquid nitrogen and then resuspended with two volumes (2 ml per gram tissue) of extraction buffer (20 mM HEPES, pH 7.5, 40 mM KCl, 1 mM EDTA, 1% Triton X-100, 0.2 mM PMSF, and 1× protease inhibitor cocktail). The protein mixtures were centrifuged at 4,000 rpm for 5 min. Then, the resulting supernatant was centrifuged at 20,000*×g* for 15 min. The supernatants were incubated with Streptavidin-agarose (S1638, Sigma, Saint Louis, MO) or an anti-Myc nanobody coupled to agarose (Myc-Trap, Chromotek, Hauppauge, NY) for 1 h at 4°C. Then, beads were washed with an extraction buffer containing 0.1% Triton X-100 and eluted with 2× SDS sample buffer (24 mM Tris-HCl, pH 6.8, 10% glycerol, 0.8% SDS, 2% 2-mercaptoethanol) containing 0.4 M urea. YFP-TbID and BZR1-MH were detected by monoclonal anti-GFP (1:2000, HT801, Transgen Biotech, Beijing, China) and monoclonal anti-Myc antibodies (1:2000, 9B11, Cell Signaling Technology, Danvers, MA), respectively. Biotinylated proteins were detected with streptavidin-HRP (1:2000, 21124, Thermo Scientific, Rockford, IL).

### Plasmids and expression of TurboID fusion proteins in plants

To generate a Gateway-compatible 35S-YFP-TbID vector, PCR fragments obtained from TurboID-containing plasmid (V5-TurboID-NES_pCDNA3) (Branon et al., 2018) and pEarleyGate101 vector were assembled by overlapping ends using Gibson assembly (NEB, Ipswich, MA). The coding sequences of *YFP*, *BIN*2, *AtSK41*, *PP2AB′α*, and *PP2AB′ɛ* in pENTR/SD/D-TOPO were subcloned into a Gateway-compatible 35S-YFP-TurboID by an LR reaction. The plasmids were transformed into *N. benthamiana* leaves transiently or into transgenic Arabidopsis plants stably. The *N. benthamiana* leaves or Arabidopsis seedlings were treated with 50 µM biotin for 3 h (unless indicated otherwise), rinsed with water, and then ground with liquid nitrogen. One gram of the tissue powder was resuspended with 1.5 ml IP buffer. After centrifugation at 12,000 rpm for 10 min, the supernatant was transferred to PD-10 desalting columns to remove free biotin. The extracts were then incubated with 30 µl Dynabead C1 Streptavidin beads at 4°C for 3 h. The beads were subsequently washed three times with a washing buffer. Biotinylated proteins were eluted by boiling the beads in 50 µl 2× SDS sample buffer and separated by SDS-PAGE. The gel blots were incubated with streptavidin-HRP to detect biotinylated proteins.

For metabolic stable isotope labeling mass spectrometry (mSIL-MS), T3 homozygous Arabidopsis seedlings of a transgenic *35S:BIN2-YFP-TbID* line and *35S:YFP-TbID* line were grown on medium containing ^14^N or ^15^N nitrogen for 16 days under continuous light before treatment with biotin for PL-MS analysis or treatment with bikinin (30 μM for 1 h) for phosphoproteomic analysis. Equal amounts (4 g) of tissues of ^14^N- and ^15^N-labeled controls and samples were mixed together and then ground in liquid nitrogen for protein extraction. For PL-MS, after extraction, desalting, and streptavidin purification, the proteins on beads were digested by Lys-C and trypsin. The peptides were fractionated into three fractions using high-pH reverse phase fractionation StageTip packed with C18 beads. For phosphoproteomics, proteins were extracted and digested into peptides. Phosphorylated peptides were enriched using IMAC. LC-MS/MS analysis was performed using a Q-Exactive HF Orbitrap mass spectrometer coupled online with an Easy-nLC 1200 (Thermo Fisher Scientific). Protein network analysis was performed in the STRING database (version 11.0) (Szklarczyk et al., 2019) using textmining, experiments, and databases, with an interaction score ≥0.7 as the cutoff. Additional interactions were added from the Arabidopsis Interactions Viewer database (Dong et al., 2019).

### Removing free biotin and affinity purification of biotinylated proteins

BIN2-YFP-TbID seedlings treated with 50 μM biotin for 3 h were ground with liquid nitrogen. One gram of the tissue powder was resuspended with 1.5 ml IP buffer (50 mM Tris-HCl, pH 7.5, 50 mM NaF, 300 mM sucrose, 1% Triton X-100) with 1× protease inhibitor cocktail (Pierce, Rockford, IL) and centrifuged at 1,500 rpm for 5 min. Resulting supernatant was centrifuged at 12,000 rpm for 10 min and each 1-ml aliquot of the supernatant was transferred to two tubes. For desalting, one of the 1-ml supernatant samples was desalted using PD-10 desalting columns (GE Healthcare, Pittsburgh, PA) according to manufacturer’s instructions and eluted with a 3-ml IP buffer. To the other supernatant sample, a 2-ml IP buffer was added to make the same volume at the desalted sample. The protein samples were incubated with 30 μl Dynabead C1 Streptavidin beads (Thermo Fisher Scientific, Waltham, MA) at 4°C for 3 h. The beads were subsequently washed three times with washing buffer (50 mM Tris-HCl, pH 7.5, 100 mM NaCl, 50 mM NaF, 0.1% Triton X-100). Biotinylated proteins were eluted by boiling the bead in 50 μl 2× SDS sample buffer (100 mM Tris-HCl, pH 6.8, 4% sodium dodecyl sulfate, 20% glycerol, and 100 mM dithiothreitol) and separated by SDS-PAGE gels (Biorad, Hercules, CA). Biotinylated protein was detected with streptavidin-HRP (21124, Thermo Scientific, Rockford, IL).

### Metabolic stable isotope labeling and affinity purification of biotinylated proteins

Metabolic stable isotope labeling (mSIL) of Arabidopsis seedlings was performed as follows. Transgenic BIN2-YFP-TbID and YFP-TbID seedlings were grown on ^14^N MS medium (1/2 MS without a nitrogen source [PhytoTechnology Laboratories], NH_4_NO_3_ [0.5 g/l, Sigma], KNO_3_ [0.5 g/l, Sigma], pH 5.7) or ^15^N MS medium (1/2 MS without a nitrogen source [PhytoTechnology Laboratories], ^15^NH_4_^15^NO_3_ [0.5 g/l, NLM-390-1, Cambridge Isotope Laboratory], K^15^NO_3_ [0.5 g/l, NLM-765-1, Cambridge Isotope Laboratory], pH 5.7) for 16 days under continuous light in a growth chamber at 22°C. ^14^N- and ^15^N-labeled seedlings were harvested and syringe infiltrated with 50 μM biotin solution. After infiltration, seedlings were transferred to 50-ml conical tubes and further incubated with 50 μM biotin for 3 h in a growth chamber. For bikinin treatment, seedlings infiltrated with 50 μM biotin solution were incubated in bikinin (30 μM bikinin and 20 μM MG132) or mock (0.1% dimethyl sulfoxide and 20 μM MG132) solution for 1 h. Four-gram tissue samples were ground in liquid nitrogen and total protein was extracted with 4 ml RIPA buffer (50 mM Tris-HCl, pH 7.5, 150 mM NaCl, 0.1% SDS, 1% Triton X-100, 0.5% sodium deoxycholate [SDC], 1 mM ethylenediaminetetraacetic acid [EDTA], 10 mM sodium fluoride, 1 mM phenylmethanesulfonyl fluoride [PMSF]) containing 1× protease inhibitor cocktail (Pierce, Rockford, IL) and a phosphatase inhibitor (PhosSTOP, Roche Applied Science, Penzberg, Germany). Protein extracts were centrifuged at 1,500 rpm for 5 min and the supernatant was recentrifuged at 12,000 rpm for 10 min. To remove free biotin, the resulting supernatant was desalted using PD-10 desalting columns (GE Healthcare, Pittsburgh, PA) according to manufacturer’s instructions. The elute was incubated with 150 μl Dynabead M-280 Streptavidin beads (Thermo Fisher Scientific, Waltham, MA) at 4°C overnight. The beads were washed with 1 ml TurboID Wash Buffer (2% SDS, 50 mM Tris pH 7.5) and 1 ml TurboID Lysis Buffer (50 mM Tris pH 7.5, 150 mM NaCl, 0.4% SDS, 1% Triton X-100, 1.5 mM MgCl_2_, 1 mM EGTA). The beads were transferred to new low protein binding tubes (Thermo Fisher Scientific) and subsequently washed with 1 ml TurboID Lysis Buffer, 1 ml of 1 M KCl, and 1 ml of 0.1 M Na_2_CO_3_. The beads were transferred to new low protein binding tubes (Thermo Fisher Scientific) and washed with 50 mM ammonium bicarbonate before tryptic digestion.

### Immunoprecipitation for mass spectrometry analysis

Proteins from 2 g tissue powder ground in liquid nitrogen were extracted with 2 ml IP buffer (50 mM Tris, pH 7.5, 50 mM NaF, 300 mM sucrose, 0.2% Triton X-100) containing 1× protease inhibitor cocktail (Pierce, Rockford, IL) and a phosphatase inhibitor (PhosSTOP, Roche Applied Science, Penzberg, Germany). Protein extracts were centrifuged at 1,500 rpm for 5 min and the supernatant was recentrifuged at 12,000 rpm for 10 min. The resulting supernatant was incubated with 50 μl GFPnanobody beads (Smart, China) at 4°C for 1 h. The beads were subsequently washed three times with IP washing buffer (50 mM Tris, pH 7.5, 100 mM NaCl, 50 mM NaF, 0.05% Triton X-100). The beads from BIN2-TbID were transferred to new low protein binding tubes (Thermo Fisher Scientific) and mixed with beads from reciprocal isotope-labeled YFP-TbID. The mixed beads were washed once with Tris washing buffer (50 mM Tris, pH 7.5) and subjected to on-bead digestion.

### On-bead digestion

Dynabeads M-280 Streptavidin or GFP nanobody beads were suspended in 100 μl of digestion buffer (12 mM sodium deoxycholate [SDC] and 12 mM sodium lauroyl sarcosinate [SLS] in 100 mM Tris-HCl [pH 8.5]) and then were 4-fold diluted using 50 mM triethylammonium bicarbonate [TEAB] buffer. The proteins bound to the beads were digested using 1 μl of Lys-C (Wako, Japan) for 3 h and then 1 μg of trypsin (Sigma, MO) for 3 h at 37°C. The supernatant solution was loaded into a StageTip packed in C18 beads (Rappsilber et al., 2007; Dimayacyac-Esleta et al., 2015) and fractionated into three fractions (200 mM ammonium formate [pH = 10.0] with 10%, 16%, and 80% of ACN) using high-pH reverse phase fractionation.

### Protein extraction and digestion for phosphoproteomics

Protein extraction and digestion were performed as previously described (Hsu et al., 2018). BIN2-YFP-TbID plants were lysed in lysis buffer (6 M guanidine hydrochloride in 100 mM Tris-HCl [pH 8.5]) with EDTA-free protease and phosphatase inhibitor cocktails. Disulfide bonds on proteins was reduced and alkylated with 10 mM Tris[2-carboxyethyl]phosphine hydrochloride (TCEP) and 40 mM 2-chloroacetamide (CAA) at 95°C for 5 min. Protein lysate was precipitated using the methanol-chloroform precipitation method. Briefly, 100 μl of lysate was added to four volumes of methanol, followed by an equal volume of chloroform with mixing. Three volumes of ddH_2_O were added to the tube with mixing. The solution was centrifuged at 16,000*×g* for 3 min. The upper aqueous layer was removed, the protein pellet was washed with four volumes of methanol, and the tube was centrifuged again. The supernatant was discarded, and the precipitated protein pellet was air-dried. Precipitated protein pellets were suspended in digestion buffer (12 mM SDC and 12 mM SLS in 100 mM Tris-HCl [pH 8.5]) and then were 5-fold diluted with 50 mM TEAB buffer. The protein amount was quantified using a BCA assay (Thermo Fisher Scientific, MA). Two milligrams of proteins from forward (^14^N-bikinin/^15^N-mock) and reverse (^14^N-mock/^15^N-bikinin) labeling were pooled and then digested with Lys-C in a 1:100 (v/w) enzyme-to-protein ratio for 3 h at 37°C, and trypsin was added to a final 1:100 (w/w) enzyme-to-protein ratio overnight. The detergents were separated from digested peptides by acidifying the solution using 10% TFA and then centrifuged at 16,000*×g* for 20 min. The digests were then desalted using a 100-mg SEP-PAK C18 cartridge (Waters, MA).

### Phosphorylated peptide enrichment and fractionation

Phosphopeptide enrichment was performed according to the reported IMAC StageTip protocol with some modification (Hsu et al., 2018). The in-house-constructed IMAC tip was made by capping the end with a 20-μm polypropylene frits disk (Agilent, CA). The tip was packed with 5 mg of Ni-NTA silica resin (Qiagen, Germany) by centrifugation at 200*×g* for 1 min. Ni^2+^ ions were removed with 100 μl of 100 mM EDTA solution. The tip was then activated with 100 μl of 100 mM FeCl_3_ and equilibrated with 100 μl of loading buffer (6% (v/v) acetic acid [AA] at pH 3.0) prior to sample loading. The digested peptides (2 mg) were reconstituted in 400 μl of loading buffer and loaded onto the IMAC tip. After successive washes with 200 μl of washing buffer (4% (v/v) AA, 25% ACN) and 100 μl of loading buffer, respectively, the bound phosphopeptides were eluted with 150 μl of 200 mM NH_4_H_2_PO_4_. The eluted phosphopeptides were loaded into a C18 beads StageTip and separated into five fractions (200 mM ammonium formate [pH = 10.0] with 6%, 10%, 14%, 18%, and 80% of ACN) using high-pH reverse phase fractionation. The fractionated phosphopeptides were dried using a SpeedVac.

### LC-MS/MS analysis

The peptides were dissolved in 5 μl of 0.3% FA with 3% ACN and injected into an Easy-nLC 1200 (Thermo Fisher Scientific). Peptides were separated on a 25-cm Easy-Spray column (75 μm ID) containing C18 resin (1.9 μm) with a column heater set at 40°C. The mobile phase buffer consisted of 0.1% FA in ultra-pure water (buffer A) with an eluting buffer of 0.1% FA in 80% CAN (buffer B) run over a linear 90 min (phosphoproteomics), 120 min (nonmodified fraction and GFPtrap pulldown), or 65 min (biotinylated and phosphorylated peptides fraction) gradient of 5%–28% buffer B at a flow rate of 300 nl/min. The Easy-nLC 1200 was coupled online with a Q-Exactive HF Orbitrap mass spectrometer (Thermo Fisher Scientific). For the identification of biotinylated proteins, the mass spectrometer was operated in the data-dependent acquisition (DDA) mode in which a full MS scan from *m*/*z* 375-1500 with the resolution of 60,000 at *m*/*z* 200. The full MS AGC value was 3 × 10^6^ with a maximum injection time (IT) of 50 ms. The 15 most intense ions being subjected to higher-energy collision dissociation (HCD) fragmentation with normalized collision energy (NCE) was set at 27%. The AGC of the fragment spectra was 5 × 10^4^ with an IT of 60 ms. The isolation width was set at 1.0 *m*/*z* and dynamic exclusion of 24 s. For bikinin phosphoproteomics analysis, the mass spectrometer was operated in the DDA with a full MS scan from *m*/*z* 375–1600. The full MS resolution was set to 60,000 at *m*/*z* 200 with an IT of 20 ms. HCD fragmentation was performed in Top10 and acquired in the Orbitrap (normalized collision energy [NCE] 27, AGC 5 × 10^4^, IT 120 ms, isolation window 1.5 *m*/*z*, and dynamic exclusion 30 s). MS2 spectra were converted to peaklist files using an in-house script PAVA, and the files were searched using Protein Prospector (version 5.20.0) (Chalkley et al., 2005) or pFind (version 3.1.5) (Chi et al., 2018) against a TAIR10 database (35,386 entries) from TAIR (https://www.arabidopsis.org/) concatenated with sequence randomized versions of each protein with a 1% FDR cutoff at the peptide level. The first peptide precursor mass tolerance was set at 10 ppm, and the MS/MS match tolerance was set at 20 ppm. For the identification of biotinylated proteins, search criteria included amino acid residues with heavy nitrogen and variable modifications of (1) oxidation on methionine residues and (2) acetylation at the N-terminus of proteins. For the identification of phosphopeptides, search criteria included a static carbamidomethylation of cysteine and amino acid residues with heavy nitrogen and variable modifications of (1) oxidation on methionine residues, (2) acetylation at the N-terminus of proteins, and (3) phosphorylation on serine, threonine, or tyrosine residues. The monoisotopic peak of the isotopic envelope was used for calculation of the peptide intensity. Relative protein expression values for each TAIR protein entry were the median value of the extracted intensity of all peptides matching to that entry. The heavy isotope incorporation efficiency was estimated using comparison of the spectra of the identified peptide with the theoretical isotope envelopes obtained from the MS-isotope module in the Protein Prospector website. The heavy nitrogen (^15^N) incorporation efficiency was set as 96% for the correction of quantification result.

### Data analysis

All data were analyzed using the Perseus software (version 1.6.2.1) (10) and Microsoft Excel. The number of unique phosphorylated peptides and phosphoproteins identified from each sample was calculated using Microsoft Excel. The median intensity ratio between light and heavy peaks (Med L/H I) was used for protein and peptide level quantitation. The average Med L/H I value of identified peptides from ^14^N and ^15^N searches was calculated. For IP-MS and PL-MS data, proteins with (1) at least two unique identified peptides and (2) the ratio of BIN2-TbID/YFP-TbID ≥3 in at least three of the four reciprocal experiments are considered BIN2-proximal proteins. The lists of BIN2-proximal proteins are shown in Supplemental Dataset 1, A–D. The IP-MS data is in Supplemental Dataset 4. For bikinin phosphoproteomics analysis, the bikinin-perturbed phosphopeptides were selected using significance A to select the outliners from the whole mock/bikinin-treated ratio controlled by *P*-value ≤0.05 in both reciprocal experiments. The lists of bikinin-perturbed phosphopeptides identified using pFind and Protein Prospecotor for data analyses are shown in Supplemental Dataset 2, A–G. Scatter plots and histograms of Log2 fold change values of identified proteins were generated by SigmaPlot (version 12.5). Gene Ontology (GO) annotation enrichment analysis was performed using PANTHER classification system (Mi et al., 2019) with Fisher’s exact test FDR ≤ 0.05 as the cutoff. The phosphorylation motif was analyzed using pLOGO (O'Shea et al., 2013). Protein network analysis was performed in the String database (version 11.0) (Shannon et al., 2003) with a high confident interaction score (a score ≥0.7) as the cutoff. Protein clusters were enriched using MCODE (version 1.5.1) (Bader and Hogue, 2003) and protein–protein interaction networks were visualized using Cytoscape (version 2.7.2) (Shannon et al., 2003).

### Data availability

The mass spectrometry proteomics data have been deposited to the ProteomeXchange Consortium (Vizcaino et al., 2014) with the dataset identification number PXD017085. (Username: reviewer43202@ebi.ac.uk, Password: EjTCR9MR). The 482 interactions have been made available at the Bio-Analytic Resource for Plant Biology's Arabidopsis Interactions Viewer 2 (http://bar.utoronto.ca/interactions2/) (Dong et al., 2019), under the DOI of this publication.

### In vitro kinase assays

To validate whether BIN2-proximal phosphoproteins are substrates of BIN2 kinase, 12 genes were cloned into the pMALc2 vector. For At5g18610 and At3g45780, a key residue in the kinase core was mutagenized to generate kinase-inactive proteins. Information about the oligomers used for cloning is presented in Supplemental Table 1. MBP-fused proteins were expressed in *E. coli* and purified using amylose beads. The in vitro kinase assays were performed as described previously (Kim et al., 2011).

## Accession numbers

Sequence data in this article can be found in the Arabidopsis Information Resource or GenBank/EMBL databases under the following accession numbers: *BZR1* (At1g75080), *BES1* (At1g19350), *BIN2* (At4g18710), *AtSK42* (At1g57870), *PP2AB′α* (At5g03470), and *PP2AB′ε* (At3g54930).

## Supplemental data

The following materials are available in the online version of this article.

**Supplemental Figure S1**. A Gateway-compatible binary vector used in this study to express YFP-tagged TurboID fusion protein.

**Supplemental Figure S2**. *BIN2-YFP-TurboID* transgenic plants.

**Supplemental Figure S3**. Bikinin causes dephosphorylation of BIN2 substrates.

**Supplemental Figure S4**. Phosphorylation site sequences of putative substrate proteins tested in vitro.

**Supplemental Figure S5**. IP-MS analysis of BIN2.

**Supplemental Figure S6**. Some peptides are targets of both *O*-GlcNAcylation and BIN2 phosphorylation.

**Supplemental Figure S7**. Venn diagram showing overlap with data published recently.

**Supplemental Table S1**. List of sequences of oligomers used for cloning.

**Supplemental Dataset S1**. List of BIN2-proximal proteins identified by TbPL-MS.

**Supplemental Dataset S2**. List of bikinin-down-regulated phosphoproteins.

**Supplemental Dataset S3**. List of *O*-GlcNAcylated GSK3 substrates in mouse.

**Supplemental Dataset S4**. Data of the anti-GFP IP-MS experiments.

## Supplementary Material

koad013_Supplementary_Data
