# Supplementary material for: Mapping the signaling network of BIN2 kinase using TurboID-mediated biotin labeling and phosphoproteomics
Source: Plant Cell. 2023 Jan 20;35(3):975–93. doi: 10.1093/plcell/koad013 (PMC10015162; doi:10.1093/plcell/koad013)
Supplement: koad013_Supplementary_Data [file koad013_supplementary_data.zip › TPC2022LSB00484Supplemental Data.pdf]

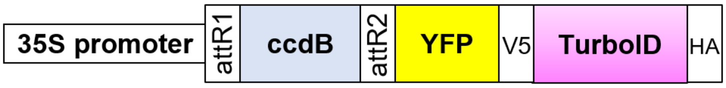

Supplemental Figure S1. A gateway-compatible binary vector used in this study to express YFP-tagged TurboID (TbID) fusion protein (*ccdB* to be replace with coding sequence of interest, such as BIN2). Supports Figure 1.

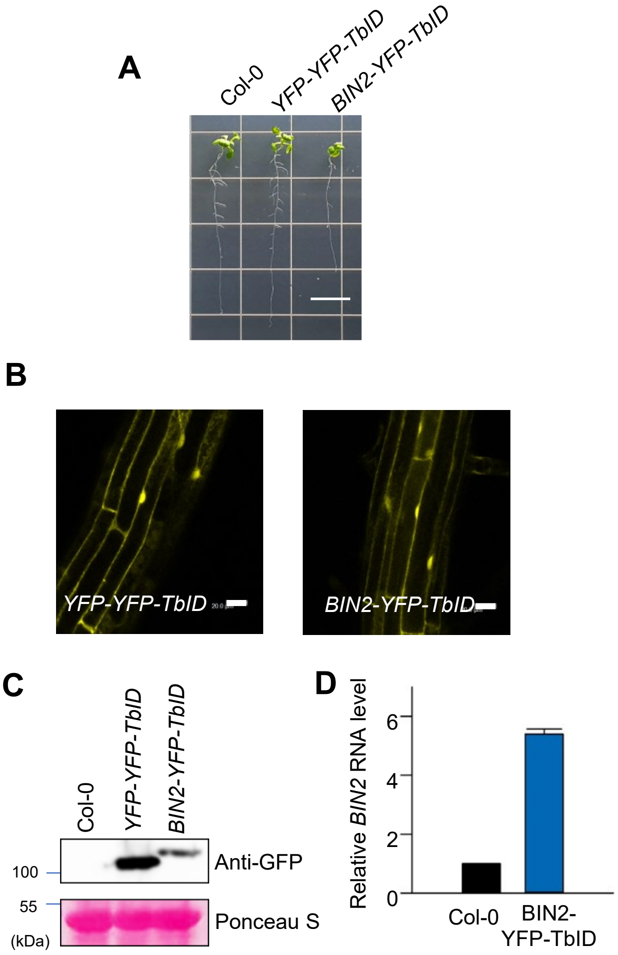

Supplemental Figure S2. Supports Figure 2. *BIN2-YFP-TurboID* transgenic plants. A, Phenotypes of the wild-type (Col-0) and transgenic Arabidopsis overexpressing *BIN2-YFP-TbID or YFP-YFP-TbID*. Seedlings were grown for 14 days on agar medium. Scale bar indicates 1 cm. B, Confocal microscopy of subcellular localization of YFP-YFP-TbID and BIN2-YFP-TbID (C) in roots of 8-d-old Arabidopsis seedlings grown MS medium in the light. Bars = 20 µm. C, Immunoblot analysis for protein expression in the seedlings shown in (A). D, RT-qPCR analysis of *BIN2* RNA level in Col-0 and *BIN2-YFP-TurboID* seedlings. Error bars show standard deviation of three biological repeats.

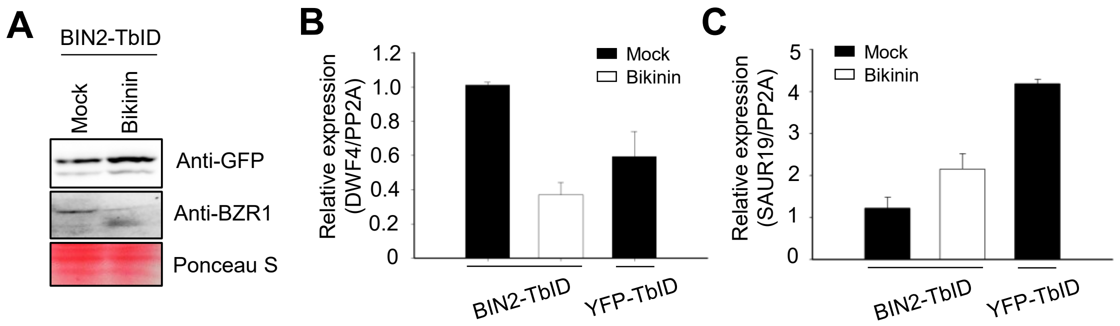

Supplemental Figure S3. Bikinin causes dephosphorylation of BIN2 substrates. Supports Figure 3.

A, Immunoblot shows BZR1 dephosphorylation (band shift) after bikinin treatment of *BIN2-YFP-TbID* seedlings. B and C, RT-qPCR results show relative expression of *DWF4* (B) and *SAUR19* (C) in *YFP-YFP-TbID* or *BIN2-YFP-TbID* seedlings treated with mock or bikinin. Columns indicate means of four repeats with S.D. The relative expression of the mock treated *BIN2-YFP-TbID* was set to 1.

TAIR	Bikinin-inhibited phosphopeptide	protein sequence	Motif
AT5G18610	SLGDG**S**SLD**pS**PAE**T**R	G**S**SLD**S**PAE**T**R	xSxxxSPxxTx
AT1G30320	T**pS**PSRLQIPDDSEFSK	N**S**SRT**S**PSRLQ	xSxxxSPxxxx
AT3G20250	RMPPQYTPSSYQVQAS**pS**PQQMSYPR	QVQAS**S**PQQMS	xxxxxSPxxxS
AT5G11710	ASAA**pS**YYSGWSMGAGSGLGK	KASAA**S**YYSGW	xSxxSxxSxx
AT5G03040	RL**pS**YPT**pS**PALPK	L**S**YPT**S**PALPK	xSxxxSPxxxx
AT3G45780	NSQNSCRS**S**GEM**pS**DGDVPGGR	S**S**GEM**S**DGDVP	xSxxxSxxxxx
AT5G64330	M**S**PSP**pS**QSMYADIPR	M**S**PSP**S**QSMYA	xSPxxSxxxxx
AT5G05970	IRDFSSTFETS**pS**TQTDNNLPS**pS**PLF**T**K	**T**FET**SS**TQ**T**DN	xxxxxSxxTxx
AT4G39680	VPEAQI**T**NSA**T**PTT**pT**PR	A**T**PTT**T**PRS**T**G	xTPxxTPxxTx
AT1G13020	GGSYSERPH**S**RAG**pS**IDE**S**R	H**S**RAG**S**IDE**S**R	xSxxxSxxxSx
AT5G18230	AF**pS**PSIVSGSQWRPG**pS**PFQ**S**QNE**T**VR	QWRPG**S**PFQ**S**Q	xxxxxSPxxSx
AT1G16860	KS**pS**GPQ**pS**GGV**T**R	S**S**GPQ**S**GGV**T**R	xSxxxSxxxTx

Supplemental Figure S4. Phosphorylation site sequences of putative BIN2 substrate proteins tested *in vitro*. Supports Figure 5. Sequences of phosphopeptides that were decreased by bikinin treatment, for the proteins tested by in vitro kinase assays shown in Figure 5. The phosphorylated Serine (pS) or Threonine (pT) and their flanking S and T residues matching the consensus GSK3 phosphorylation motif (S/TxxxS/T) are marked in bold.

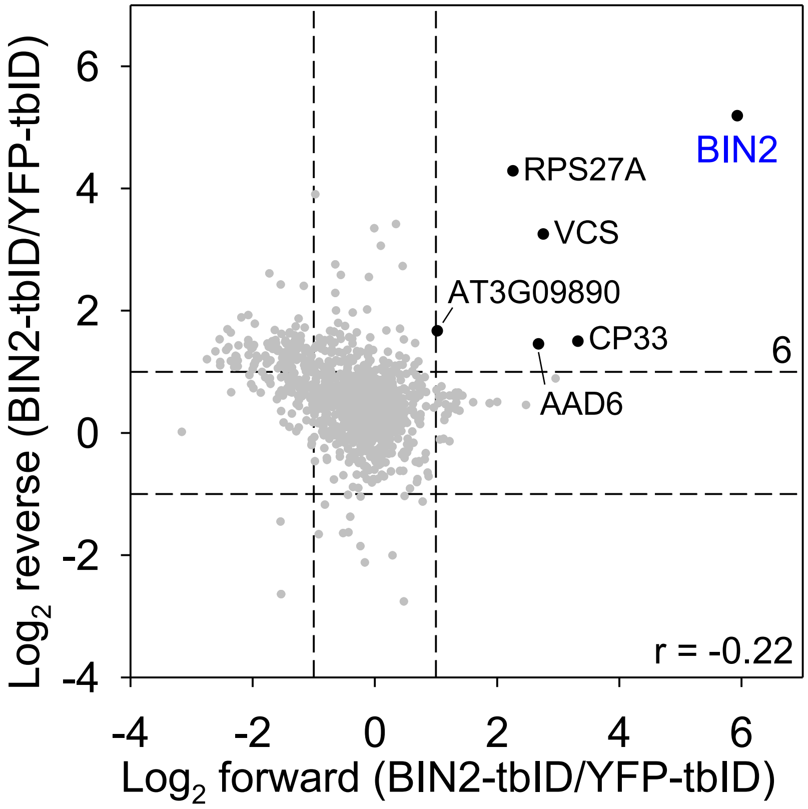

Supplemental Figure S5. IP-MS analysis of BIN2. Supports Figure 3. Aliquots of the protein extracts used in the PL-MS experiments, shown in Figure 3B, were immunoprecipitated using anti-GFP nanobody beads. The chart shows the ratios between BIN2-YFP-TurboID and YFP-YFP-TurboID from two reverse-labeling replicates (forward and reverse). Six proteins including BIN2 showed ratios >2 in both replicates.

Protein	Peptide sequence
OXS2	^{500}F**T**DSALASAVFSPTHK^{515}
TZF8	^{506}FSDQLAVSSVLSPSHK^{520}
PUM5	^{325}RMPPQYTPSSYQVQASSPQQMSYPR^{349}
ECT5	^{244}HSSSISPALSPQPLGSYGSYGQNIPMGSQR^{273}

Supplemental Figure S6. Some peptides are targets of both O-GlcNAcylation and BIN2 phosphorylation. Supports Figure 6. The peptides were identified among both O-GlcNAcylated peptides and BIN2-dependent phosphopeptides. Phosphorylated residues are shown by underline. O-GlcNAc modified residue of OXS2 is bold (site not defined for other proteins).

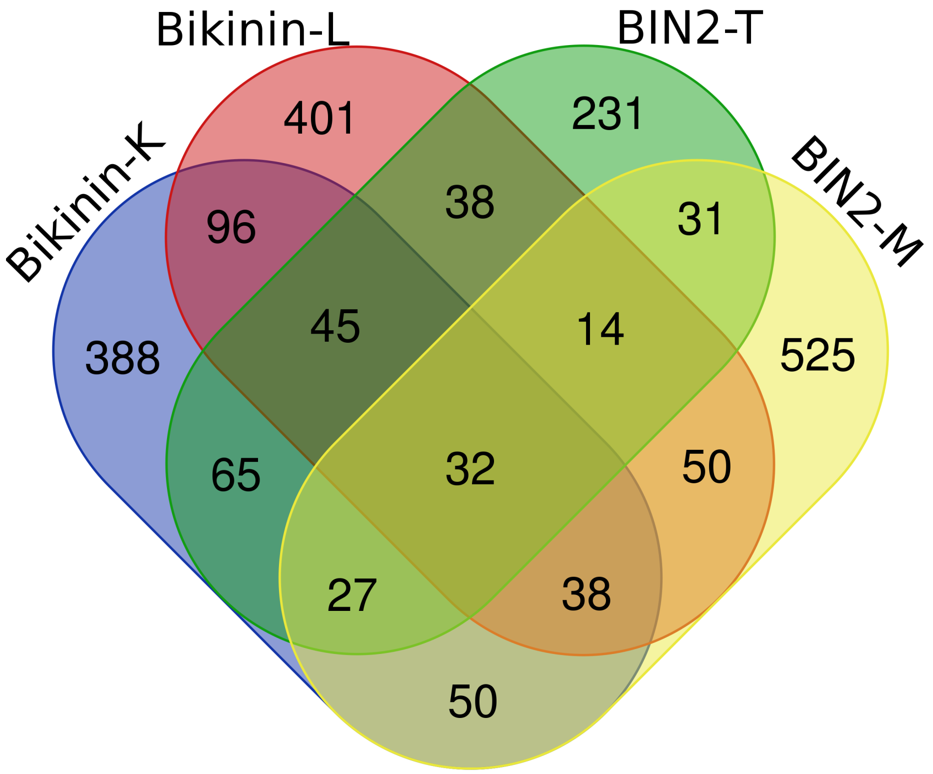

Supplemental Figure S7. Venn diagram showing overlap with data published recently. Supports Figure 6. The Venn diagram compares the bikinin-inhibited phosphoproteins identified in this study (Bikinin-K) and by Lu et al (2022)(Bikinin-L), BIN2-proximal protein identified by BIN2-TurboID (BIN2-T) in this study, and BIN2 *in vitro* substrates (BIN2-M) identified by Montes et al. (Supplemental Dataset 8e of Montes et al., 2022).

Supplemental Table S1. List of oligomers used for cloning

AGI Number	Name	Sequence (5'→3')
**For cloning**		
At3g45780 (PHOT1)	*PHOT1-Kpn1-F* *PHOT1-EcoR1-R*	CGGGGTACCATGGAACCAACAGAAAAACC CGGGAATTCGTAAAAACATTTGTTTGCAG
At5g64330 (NPH3)	*NPH3-BamH1-F* *NH3-EcoR1-R*	CGCGGATCCATGATGTGGGAATCTGAGAG CGGGAATTCGTTGAAATTGAGTTCCTCCATCG
At1g16860 (SHOU4L$^{1-209}$)	*SHOU4L-BamHI-F* *SHOU4L-XhoI-R*	CGCGGATCCATGGGTTCGAGATACCCATC CCGCTCGAGTGAGGCTTTGGAAAGCTCTTCAG
At5g11710 (EPS1)	*EPS1-EcoRI-F* *EPS1-XhoI-R*	CCGGAATTCATGGATTTCATGAAGGTCTTCG CCGCTCGAGTGCTGCTTAAAGCCACCAGATTG
At3g09840 (CDC48)	*CDC48-KpnI-F* *CDC48-NotI-R*	CGGGGTACCATGTCTACCCCAGCTGAATC AAGGAAAAAAGCGGCCGCGAATTGTAGAGATCAT CATCGTC
At4g39680 (ACINUS)	*ACINUS-BamHI-F* *ACINUS-XhoI-R*	CGCGGATCCATGTCGTCATCGCCTTTTCCAG CCGCTCGAGTGCTTGTTATTATTCGCTGCAAG
At1g13020 (eIF4B2)	*eIF4B2-KpnI-F* *eIF4B2-NotI-R*	CGGGGTACCATGTCGAAACCTTGGGGTGGA AAGGAAAAAAGCGGCCGCGACCATCCTTCCCTTGC TGACGA
At3g20250 (PUM5)	*PUM5-KpnI-F* *PUM5-NotI-R*	CGGGGTACCATGACGACTACGCAGAGCGCT AAGGAAAAAAGCGGCCGCGAACCTTCTGTTCCCTC TTCTGAG
At5g18230 (NOT3)	*NOT3-KpnI-F* *NOT3-Not1-R*	CGGGGTACCATGGGTGCGAGCCGGAAATT AAGGAAAAAAGCGGCCGCGATACGACGAGTTCAT CTTCAAG
AT5G03040 (IQD2)	*IQD2-EcoR1-F* *IQD2-Not1-R*	AGGAATTCATGGGGAAAAAAGCTAAATG ATAGTTTAGCGGCCGCGAGCTGCCTGCTCCGTT GGT
AT5G05970 (NEDD1)	*NEDD1-Kpn1-F* *NEDD1-Not1-R*	AGGGTACCATGATGTCGAACTTGGTAG ATAGTTTAGCGGCCGCGACTAAAGCCTTTGTCT GAGC
At1g30320	*At1g30320-Kpn1-FAt1g30320-Xho1-R*	GAGGTACCATGGATTACGAGAGGATACA CCACTCGAGTGTGAGAACCAACCACAACA
At5g18610	*At5g18610-Kpn1-F* *At5g18610 Not1-R*	GCTCGGAATTCATGAGTGGGTGTTTGCCTTG ATAAGAATGCGGCCGCGAGTCATTTGTACTATCA
**For mutagenesis**		
At3g45780 (*PHOT1*-D806N)	*Phot1*-D806N-F *Phot1*-D806N-R	GATATCTCTTTGTCGAATTTTGATCTGTC GACAGATCAAAATTCGACAAAGAGATATC
At5g18610 (At5G18610-K112R)	At5G18610-K112R-F At5G18610-K112R-R	GATAGTAGCTGTTCGACAGCTTGATCGA TCGATCAAGCTGTCGAACAGCTACTATC
**For RT-qPCR**		
At4g18710 (*BIN2*)	*BIN2*-qRT-F *BIN2*-qRT-R	TCTGCTGGTTGTGTTCTTGC AAGATCTTGTGCCAGGGATG
