# Supplementary material for: Mapping the signaling network of BIN2 kinase using TurboID-mediated biotin labeling and phosphoproteomics
Source: Plant Cell. 2023 Jan 20;35(3):975–93. doi: 10.1093/plcell/koad013 (PMC10015162; doi:10.1093/plcell/koad013)
Supplement: koad013_Supplementary_Data [file tpc22.00484PeerReviewReport.pdf › tpc22.00484PeerReviewReport.pdf]

Mapping the signaling network of BIN2 kinase using TurboID-mediated biotin labeling and phosphoproteomics

Tae-Wuk Kim, Chan Ho Park, Chuan-Chih Hsu, Yeong-Woo Kim, Yeong-Woo Ko, Zhenzhen Zhang, Jia-Ying Zhu, Yu-Chun Hsiao, Tess Branon, Krista Kaasik, Evan Saldivar, Kevin Li, Asher Pasha, Nicholas J Provart, Alma L Burlingame, Shou-Ling Xu, Alice Y Ting, Zhi-Yong Wang

Corresponding author: Zhi-Yong Wang zywang24@stanford.edu

Review timeline:

**TPC2022-LSB-00484**	Submission received:	May 25, 2022
	1st Decision:	July 23, 2022 *revision requested*
**TPC2022-LSB-00484R1**	1st Revision received:	Sept. 17, 2022
	2nd Decision:	Oct. 12, 2022 *accept with minor revision*
**TPC2022-LSB-00484R2**	2nd Revision received:	Nov. 23, 2022
	3rd Decision:	Dec. 1, 2022 *acceptance pending, sent to science editor*
	Final acceptance:	Jan. 13, 2023

REPORT: (The report shows the major requests for revision and author responses. Minor comments for revision and miscellaneous correspondence are not included. The original format may not be reflected in this compilation, but the reviewer comments and author responses are not edited, except to correct minor typographical or spelling errors that could be a source of ambiguity.)

TPC2022-LSB-00484 1st Editorial decision – *revision requested* **July 23, 2022**

(Following up from Plant Biology - thanks for sending your manuscript to The Plant Cell!)

We have received reviews of your manuscript entitled "Mapping the signaling network of BIN2 kinase using TurboID-mediated biotin labeling and phosphoproteomics." Thank you for submitting your best work to The Plant Cell. The editorial board agrees that the work you describe is substantive, falls within the scope of the journal, and may become acceptable for publication, pending revision and potential re-review. When preparing a revised version, please consider and give clarification of all the comments from reviewers.

--- Reviewer comments:

[Reviewer comments shown below along with author responses]

TPC2022-LSB-00484R1 1st Revision received **Sept. 17, 2022**

Reviewer comments and **author responses**:

Reviewer #1:

GSK3 signaling network controls diverse developmental and acclimation processes in plants. However, the targets of BIN2 are mainly unknown. In this manuscript, the authors use the Turbo-ID based proximity labeling approach and quantitative phosphoproteomics to acquire high-quality phosphonetwork of BIN2 kinase. The study also discovered a significant overlap between the GSK3 phosphorylome and the O-GlcNAcylome, which, as far as I know, has not been reported yet in plants. The experiments in this manuscript are well-designed, and the data and conclusion are convincing. This study uses a new approach to resolving a long-stand question in plant biology. In my opinion, this manuscript should be considered in top-tier journal like The Plant Cell.

1. In the third section, Co-regulation by GSK3 and O-GlcNAc is an ancient feature of cellular regulation, the authors discussed the potential relationship between O-GlcNAc and GSK3-mediated phosphorylation. I agree with the author

this point is interesting. However, as lacking experimental evidence, some conclusions need to be revised to avoid overstating.

> **Response: We have tuned down our conclusions. We changed the subtitle to: "GSK3 and O-GlcNAc target overlapping proteomes in plants and animals".**

2. For TbPL-MS analysis, the author chose a mild dwarf phenotype BIN2-TurboID plants, which indicate moderate expression levels. It's better to use the BIN2 antibody or qRT-PCR to confirm the level of BIN2 in WT and BIN2-TurboID plants.

> **Response: We have performed qRT-PCR and added text (line 134): Quantitative RT-PCR showed that the *BIN2-YFP-TbID* plants expressed about 4-fold more *BIN2* RNA than wild type (Supplemental Figure S2D). However, a smaller increase is expected for BIN2 protein, which is posttranslationally regulated by ubiquitination and degradation (Zhu *et al.*, 2017).**

3. Line 173, "Supplemental Figure S4, A-C" should be "Supplemental Figure S3".
4. Line 203, "Figure 4D" should be "Figure 4E"

> **Responses: We have fixed these errors. Thank you.**

Reviewer #2:

BIN2, as a member of the GLYCOGEN SYNTHASE KINASE 3 (GSK3) family protein, plays an important role in plant developmental and stress acclimation processes. Identification of BIN2 substrate is of great scientific significance for comprehensive understanding of the molecular mechanisms and regulatory network of BIN2. Given that the interaction between kinase and substrate is often transient and dynamic, the strength of the interaction is usually weak. Therefore, traditional IP-MS methods are often not ideal for identifying the above-mentioned types of interactions. The recently emerged TurboID-based proximity labeling (TbPL) technology provides a new approach for studying the interaction between kinases and substrates. Here, Kim et al. used TbPL to analyze the BIN2's proximal proteins. They identified more than 400 potential BIN2 substrates. Meanwhile, in order to further confirm the reliability of the above results, the author further identified the proteins that depend on BIN2 for phosphorylation through quantitative phosphoproteomics. The results showed that more than one third of BIN2 proximal proteins possess phosphorylation modification. The author also selected some BIN2 proximal proteins for in vitro kinase analysis. Moreover, the authors also performed signaling network analysis of the identified BIN2 proximal proteins, and showed that the identified BIN2 proximal proteins are associated with multiple key cellular processes. Intriguing, the authors found that the phosphorylation modification of BIN2 proximal proteins is correlated to O-GlcNAc modification, suggesting a close relationship between the GSK3 pathway and the nutrient-sensing O-glycosylation pathway. All these results further substantiate the robustness of TbPL in studying transient or weak interactions like kinase and substrate, and also provide new scientific evidence for in-depth understanding of the molecular mechanism involved in GSK3 family proteins. The experimental design of the article is generally good, and the writing of this manuscript is easy to follow. Some issues need to be addressed before it is acceptable for publication in the Plant Cell.

1. line 167, Can the authors explain why quantitative phosphoproteomics analysis was performed with bikinin inhibitor treatment but not with bin2-1 Arabidopsis mutant plants?

> **Response: The bin2-1 is a hypermorphic mutant that accumulates a high level of BIN2 proteins. The constitutive BIN2 activity causes cumulative proteomic changes that include direct and indirect (e.g. via transcriptomic changes) effects of BIN2.**

2. line 153, Why set 3-fold as the cut-off instead of 2-fold?

> **Response: TurboID is very sensitive and can biotinylate proteins within 10 nm proximity. All known BIN2 interacting proteins and substrates in the BR pathway were enriched over 3-fold.**

3. Fig. 1, panels B-D, Usually, the Ponceau S loading control is generally placed at the bottom of the Input test; In addition, panel C, Pull down/streptavidin test results show that the last lane does not seem to be the result from the same membrane, please provide the original data or re-perform this experiment.

Response: We think it's better to keep the images to be compared together. The loading control can be either at the top or bottom, but would not be good if placed in the middle between blots.

Panel C is the original image, as shown below.

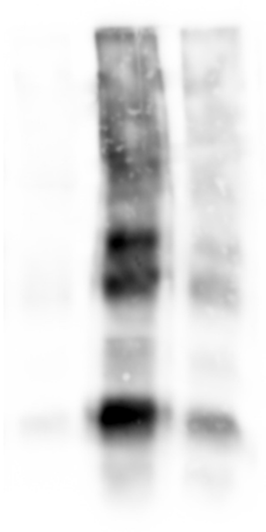

4. Fig. 2C, Biotin （uM), "uM" should be "µM". In the legend. "Equal aliquots of rotein" should be "Equal aliquots of protein".

Response: Thank you. We have fixed these errors.

5. Supplemental Figure S2B, left panel, YFP-YFP-TbID fluorescence is shown to enter the nucleus, is it reasonable? Figure S2C, "Kda" should be "kDa"

Response: YFP-YFP-TbID is localized in both cytoplasm and nucleus. The protein size (Figure S2C) is around 100 kDa. Maybe the three tandem fusion form a rod or beads-on-string shape and thus can diffuse into the nucleus.

We have changed all Kda to kDa. Thank you.

6. Supplemental Figure S4A, Fig. 4C and 4D showed some conserved motifs that are related to the phosphorylation, I wonder whether there are similar conserved motifs within the proteins tested in Figure S4A.

Response: Thank you for the suggestion. We listed the bikinin down-regulated phosphoproteins tested in Figure S4A (We moved Figure S4 (in vitro kinase assays) to Figure 5) and their phosphorylation motifs in the new Supplemental Figure 4. Most phosphoproteins tested in Figure S4A have the conserved motifs: (S/T)xxx(S/T)P or (S/T)Pxx(S/T) shown in Figure 4C.

7. Supplemental Figure S5, What does blue-colored Hsp90 indicate in the diagram? I suggested marking the points corresponding to 6 proteins with color and directly indicate the name of those 6 proteins.

Response: We thank the reviewer for the suggestion. We have modified Figure S5 according to the reviewer's suggestion.

<u>Reviewer #3</u>:

BIN2 is the best characterized GSK3 family protein in plant and is critical negative regulator of BR signaling. Here Kim et al use several proteomics methods to identify BIN2 substrates. They first use TurboID to identify proteins proximal to BIN2, which is main strength of the manuscript. They then identify phosphopeptides that decrease following treatment with GSK3 inhibitor bikinin. Interestingly by overlapping with a previous report many BIN2 substrates are O-GlcNAc modified - the functional relevance of this observation is unclear. Overall, the identification of many BIN2 substrates fills a major gap in the understanding of BIN2 signaling.

1) The abstract and introduction suggest that this study is the first to show TurboID works for kinases. This has been shown before by Arora et al published in Plant Cell in 2020 but was not cited.

Response: We added in the Introduction (Line 87): "TurboID has also been shown to detect various protein-protein interactions in several plant model systems (Arora et al., 2020)."

Arora et al. detected interaction between two RLKs in a receptor kinase complex but did not identify kinase substrates. Our focus is on the transient interactions between a kinase and its substrates.

2) Bikinin proteomics appear to be done using only two replicates which is underpowered and limits the data as a resource.

Response: Metabolic stable isotope labelling mass spectrometry is considered the most accurate quantitative mass spectrometry method. Because the sample and control are mixed at the beginning of sample preparation, technical variations are avoided. Two replicates are accepted as sufficient for this proteomic method in the proteomic field. The quality of the bikinin proteomic data is evident from the high overlap with the BR-responsive phosphoproteomic data - 90% (151 of 169) of the bikinin-responsive phosphoproteins were BR responsive.

3) In Results the Nicotiana experiments clearly state which promotor was used. Please clarify in the subsequent sections that Arabidopsis stable expression was driven by 35S. Listing the promoter in construct name throughout would also help clarity - Plant Cell has a suggested convention: https://academic.oup.com/plcell/pages/general-instructions Section: "Gene fusions, constructs, and mutations"

Response: We have added 35S promoter in the Results section.

4) What generation of transgenic plants used? Were experiments done from a single transgenic line or were multiple independent lines used (either is fine)?

Response: We have added in Material and Methods: T3 homozygous Arabidopsis seedlings of a transgenic *35S:BIN2-YFP-TbID* line and *35S:YFP-TbID* line were grown on medium

5) Define what the dashed lines designate in Figure 3b legend.

Response: We thank the reviewer's suggestion. The dashed lines mean the cut-off ratio (3 fold) of significantly enriched biotinylated proteins in two reciprocal replicates. We modified the Figure 3b and added the description in the legend.

6) In Line 173 is reference for Supplemental Figure 3?

Response: We have fixed this error. Thank you.

7) How were the data normalized?

Response: We used the median intensity ratio (Med L/H I) calculated by Protein Prospector to quantify the amount of light (14N) and heavy (15N)-labeled biotinylated proteins and enriched phosphopeptides. More detailed information is in Data Analysis section.

8) Right now the supplemental tables are incomplete and only list differentially expressed proteins with no quantitative values. Listing all detected proteins/phosphopeptides with their values in each replicate, the calculated ratios and statistical test result will be useful as resource.

Response: We thank the reviewer's suggestion. We added supplemental table S5-S12 to the supplemental materials. The tables list all the enriched proteins from IP-MS (Table S5 and S6) and PL-MS data (Table S7 and S8) and phosphopeptides identified from data analyzed by Protein Prospector (Table S9 and S10) and pFind (Table S11 and

S12) with their normalized ratios in reciprocal labeling replicates.

TPC2022-LSB-00484R1 2nd Editorial decision – *accept with minor revision* Oct. 12, 2022

We have received reviews of your manuscript entitled "Mapping the signaling network of BIN2 kinase using TurboID-mediated biotin labeling and phosphoproteomics." On the basis of the advice received, the board of reviewing editors would like to accept your manuscript for publication in The Plant Cell. This acceptance is contingent on revision based on the comments of our reviewers.

In particular, please consider the following: Montes et al. 2022 (New Phytologist, doi.org/10.1111/nph.18404) reported the identification of BIN2 substrates using global in vitro kinases assays. We suggest that you please cite this paper and add some comparison with their data in the discussion section of the manuscript.

Please highlight all changes and include a detailed annotation of changes of the text, with line numbers, and noting your responses to the comments.

TPC2022- LSB-00484R2 2nd Revision received Nov. 23, 2022

Reviewer comments and **author responses**:

Editor:

In particular, please consider the following: Montes et al. 2022 (New Phytologist, doi.org/10.1111/nph.18404) reported the identification of BIN2 substrates using global in vitro kinases assays. We suggest that you please cite this paper and add some comparison with their data in the discussion section of the manuscript.

Thank you for the suggestion. Done.

Please highlight all changes and include a detailed annotation of changes of the text, with line numbers, and noting your responses to the comments.

We submitted a copy with changes tracked.

Reviewer #1:

The authors did an excellent job of revising the manuscript, and I'm no more concern about it. In addition, I would like to clarify that I agree with the author's response to reviewer 3 comments 2, two reciprocal labeling replicates of stable isotope labeling are a standard method in the proteomic field.

We appreciate the support and clarification.

Reviewer #2:

The authors have addressed my concerns and I have no further comments on this work.

Thank you.

Reviewer #3:

The authors have done a nice work addressing some but not all of my comments.

The functional relevance of BIN2 substrates and O-GlcNAc overlap remains unclear.

Yes, this will be a very interesting subject for future study.

The use of 2 replicates for bikinin experiment does limit the data as a resource. While metabolic stable isotope labeling is among the most accurate quantification methods this does not factor into accounting for biological variance. With 2 replicates there will be high false-negative rates - it is not known how many of the bikinin non-response phosphoproteins are actually bikinin responsive. This is why omics fields have moved away from 2 replicate experiments. Perhaps stating that the bikinin experiment is a preliminary screen and not an exhaustive resource of bikinin response is okay.

Readers understand that no proteomic study provides complete coverage of the biological system. What is preliminary or exhaustive is subjective. Our bikinin dataset is better than that reported by Lu et al., 2022 based on the overlap with BIN2-proximal proteins or with BIN2 in vitro substrates (Supplemental Figure 7). However, we do not think it is necessary to state whether our bikinin experiment is preliminary or exhaustive.

The additional supplement tables are helpful but having multiple tables from different analysis tools with replicates separate and only listing the perturbed phoshopeptide from a single replicate is difficult to navigate. For example, providing a single table of the bikinin experiment with all replicate values normalized, with the actual statical test result for all phosphopeptides.

Thank you for the suggestion. We added Supplemental Datasets 1B, 2B and 2C, each combining replicate datasets. We moved previous Datasets (Tables) 7 and 8 to Datasets 1C and 1D; Datasets 9-12 to 2D-G; Datasets 5 and 6 to Datasets 4A and 4B. Thus, the quantitation data from replicate experiments are now together in one excel file.

TPC2022-LSB-00484R2 3rd Editorial decision – *acceptance pending* **Dec. 1, 2022**

We are pleased to inform you that your paper entitled "Mapping the signaling network of BIN2 kinase using TurboID-mediated biotin labeling and phosphoproteomics" has been accepted for publication in The Plant Cell, pending a final editorial review by a science editor.

Final acceptance from Science Editor **Jan. 13, 2023**
